# Interfacial Polarization Dynamics of Nitrile Dipoles in Organic Synaptic Transistors for Acoustic Reservoir Computing

**DOI:** 10.1002/advs.78389

**Published:** 2026-10-20

**Authors:** Yutian Liu, Jiale Huang, Haoran Fang, Jichen Zhao, Fan Ye, Bo Huang, Ruibin Liang, Jintao Xu, Fanqi Wang, Mingjun Huang, Zhiming Lin, Shuguang Zhang, Linfeng Lan, Junbiao Peng

**Affiliations:** ^1^ State Key Laboratory of Luminescent Materials and Devices Guangdong Basic Research Center of Excellence for Energy and Information Polymer Materials South China University of Technology Guangzhou China; ^2^ South China Advanced Institute for Soft Matter Science and Technology School of Emergent Soft Matter South China University of Technology Guangzhou China; ^3^ School of Advanced Materials and Technology Guangzhou Polytechnic University Guangzhou China; ^4^ Department of Rheumatology The Third Affiliated Hospital of Sun Yat‐Sen University Guangzhou China

**Keywords:** acoustic temporal processing, interfacial dipolar relaxation, organic synaptic transistors, polyacrylonitrile interlayer, reservoir computing

## Abstract

Polar polymer interlayers have emerged as core functional layers in organic synaptic transistors (OSTs), enabling diverse neuromorphic behaviors through interfacial dipolar polarization and relaxation. However, the interfacial dipolar mechanisms governing memory window (ΔV) formation and modulation remain insufficiently understood. Here, polyacrylonitrile (PAN) is selected as a representative polar polymer interlayer to exploit nitrile (C≡N) dipolar relaxation for temporal acoustic reservoir computing (RC). It is found that the ΔV varies nonmonotonically with PAN concentration, suggesting a dipolar competition mechanism between interfacial polarization strength and dipole relaxation capability. Accordingly, dipolar relaxation underpins multiscale synaptic responses and fading memory for temporal RC. The device further exhibits stable and symmetric weight updates, with a low asymmetry ratio (AR) of 5.06% and cycle‐to‐cycle variations (CCVs) below 1%. Notably, the PAN‐OST operates with an ultralow event energy of 50 aJ while maintaining a high signal‐to‐noise ratio (SNR) of 50.47 dB. The PAN reservoir achieves 97.17% accuracy in spoken digit recognition and a maximum accuracy of 94.8% in the more challenging speech emotion recognition. Overall, this work establishes an interfacial polarization‐driven pathway for energy‐efficient neuromorphic computing and next‐generation intelligent hardware.

## Graphical abstract

Interfacial nitrile dipoles unlock tunable memory in organic synaptic transistors through competing polarization and relaxation. Their finite time dynamics are directly harnessed for acoustic reservoir computing, delivering 97.17% spoken digit recognition, reliable speech emotion and snoring recognition, and an ultralow event energy of only 50 aJ.

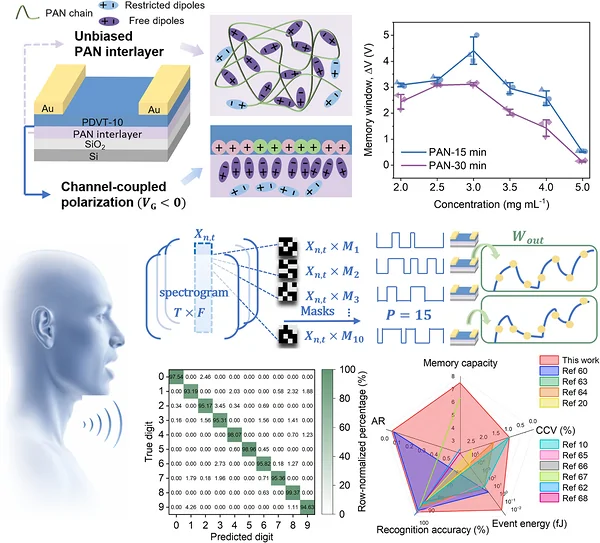

## 1 Introduction

As artificial intelligence (AI) is increasingly deployed in edge sensing, there is a growing demand for sensory systems that can perceive and process dynamic real‐world information with low power consumption. These systems play important roles in human–machine interaction, autonomous robotics, and health monitoring [1, 2, 3]. Among various sensory modalities, acoustic signals are among the most ubiquitous temporal inputs. They encode rich information related to speech communication, emotional expression, and physiological states [4, 5, 6]. Effective acoustic recognition requires the extraction of discriminative temporal and spectral features from acoustic representations such as spectrograms. In this process, transient features, preceding information, and linguistic content jointly determine the recognition outcome [7, 8, 9]. However, the separation of memory and processing in conventional von Neumann architectures requires frequent data transfer, increasing latency and energy consumption in edge systems [10]. Therefore, energy‐efficient neuromorphic hardware that can locally process temporal acoustic features while retaining information from preceding inputs is highly desirable for edge sensory intelligence.

This requirement for capturing temporal correlations naturally aligns with physical reservoir systems, where history‐dependent dynamics retain information from previous inputs and transform temporal correlations into distinguishable transient states. Reservoir computing (RC) provides an efficient neuromorphic framework for temporal information processing [11]. In RC, a nonlinear dynamical reservoir maps temporal inputs onto transient states in a high‐dimensional space, improving their separability for a simple linear readout [12, 13]. With the reservoir typically fixed and only the readout trained, RC reduces both training costs and computational complexity [14]. In physical RC, these reservoir states can be generated directly from the intrinsic dynamics of devices [15]. The required nonlinear transformation and fading memory can arise from various device‐intrinsic relaxation processes, including volatile memristive dynamics [16, 17], ion redistribution [18], electrochemical doping/de‐doping [19], ferroelectric polarization [20], and interfacial dipolar polarization [21]. Consequently, understanding the physical origin of these history‐dependent dynamics and their connection to device‐level relaxation mechanisms is essential for designing reliable physical reservoirs.

Three terminal synaptic transistors are promising device platforms for neuromorphic hardware. They spatially separate the gate input from the source drain current readout, reducing crosstalk [22]. Gate voltage pulses modulate the channel current, thereby mimicking synaptic weight updates. Volatile current dynamics provide short‐term plasticity (STP) and fading memory required for RC, whereas persistent current responses support long‐term potentiation/depression (LTP/LTD) [23, 24, 25]. Among all kinds of synaptic transistors, organic synaptic transistors (OSTs) offer distinct advantages, including solution processability, mechanical flexibility and molecular tunability [26]. In OSTs, the interface between the dielectric and semiconductor is critical because charge carriers are accumulated and transported near this interface [27, 28]. At this interface, a polar interlayer can retain polarization after gate bias stimulation, thereby producing history‐dependent carrier accumulation in the channel [29]. In the transfer characteristics, this effect manifests as an enhanced reverse sweep current, resulting in a pronounced memory window (ΔV) [30].

Recent studies have further demonstrated that engineering gate‐dielectric composition and polarization can effectively modulate transistor characteristics and enable low‐energy synaptic operation [31]. Polar interlayers in OSTs can be broadly classified into ferroelectric layers and nonferroelectric polar polymer layers. In ferroelectric systems, ΔV is closely associated with ferroelectric polarization [32]. It generally increases with remanent polarization in the low polarization regime but tends to approach a limit governed by the coercive field and ferroelectric layer thickness at higher polarization [33, 34]. Compared with ferroelectric systems, nonferroelectric polar polymers can enable lower‐voltage operation and more tunable volatile relaxation dynamics [35]. In these systems, ΔV is strongly influenced by interfacial dipolar effects, which are related to polymer polarity, active polar group density, dipole orientation, relaxation kinetics, and interfacial microstructure [36, 37, 38, 39, 40, 41]. Baeg et al. suggest that increasing polymer polarity can facilitate charge dissipation, thereby reducing the ΔV [37]. Park et al. report that a higher density of active polar groups enlarges the ΔV [38]. Tsai et al. show that the orientation of polar groups contributes to ΔV formation [39]. Together, these findings indicate that the ΔV reflects the evolution of an interfacial dipolar state under electrical stimulation. However, the formation and modulation of this state, as well as its role in fading memory and reservoir dynamics, remain unclear.

Here, we develop OSTs with tunable ΔV based on controllable nitrile (C≡N) interfacial dipoles. An ultrathin polyacrylonitrile (PAN) interlayer is introduced between the SiO_2_ gate dielectric and the poly[3,6‐dithiophen‐2‐yl‐2,5‐bis(2‐decyltetradecyl)pyrrolo[3,4‐c]pyrrole‐1,4‐dione‐alt‐thienylenevinylene‐2,5‐yl] (PDVT‐10) semiconductor to incorporate C≡N dipolar units. We propose a dipolar competition between polarization strength and relaxation capability. This competition underpins synaptic responses and plasticity over multiple timescales, including excitatory postsynaptic current (EPSC), paired‐pulse facilitation (PPF), STP, LTP/LTD, and learning‐forgetting‐relearning behavior. These nonlinear and history‐dependent dynamics enrich transient reservoir states and improve the separability of temporal acoustic patterns. Leveraging the experimentally derived PAN dynamics, we construct a compact RC framework with task‐specific readouts for spoken digit recognition, speech emotion recognition, and snoring detection. Compared with the baseline models, the PAN reservoir framework achieves higher recognition accuracies across temporal acoustic tasks. These results identify dipolar competition as the origin of the nonmonotonic evolution of ΔV and demonstrate the conversion of interfacial dipolar relaxation into RC capability for low power edge acoustic intelligence.

## 2 Results and Discussion

### 2.1 Design Principles of PAN‐OSTs

To impart temporal encoding capability to the OST, an ultrathin PAN interlayer was inserted between the SiO_2_ gate dielectric and the PDVT‐10 semiconductor channel. As shown in Figure S1, PAN contained pendant C≡N groups with large dipole moments, which served as dipolar units within the interlayer. Under gate bias, these dipolar units were expected to undergo polarization and relaxation, contributing to history‐dependent modulation of the PDVT‐10 channel current. Figure 1a illustrates the operating mechanism of the PAN‐OST. During the forward sweep from 5 to −5 V, the negative gate bias promoted hole accumulation in the p‐type PDVT‐10 channel, accompanied by a gradual buildup of polarization in the PAN interlayer. When the gate voltage was swept back from −5 to 5 V, the established polarization did not immediately return to its initial state. The retained polarization therefore enhanced hole accumulation during the backward sweep, resulting in a larger absolute drain current (|*I*_D_|) at the same gate voltage (*V*_G_), thereby producing a ΔV in the transfer curve (Figure 1b) [42]. The ΔV was extracted at |*I*_D_| = 7 nA throughout this work. This current level provided reliable signal extraction and enables consistent comparison of ΔV among different devices. ΔV was defined as the gate voltage difference between the forward and backward sweeps (ΔV = *V*_1_ − *V*_2_). The representative device exhibited a ΔV of 3.0 V, which quantified the hysteresis and reflected the history‐dependent response associated with synaptic memory [43]. To exploit this response for temporal state encoding, a sequence of voltage pulses was applied to the gate. As shown in Figure 1c, repeated negative gate pulse stimulation induced a cumulative evolution of |*I*_D_|, rather than producing only isolated responses to individual pulses. The partial relaxation between adjacent pulses indicates the retention of preceding input information, enabling temporal integration in the channel current response. To assess this temporal mapping capability, four‐bit gate pulse sequences were applied to the device. The resulting current responses were code‐dependent and clearly distinguishable (Figure 1d), confirming the effective separation of different pulse sequences. Based on the fitted rise and decay dynamics, the corresponding 16‐state map was constructed (Figure 1e), which defined the reservoir space.

**FIGURE 1:**
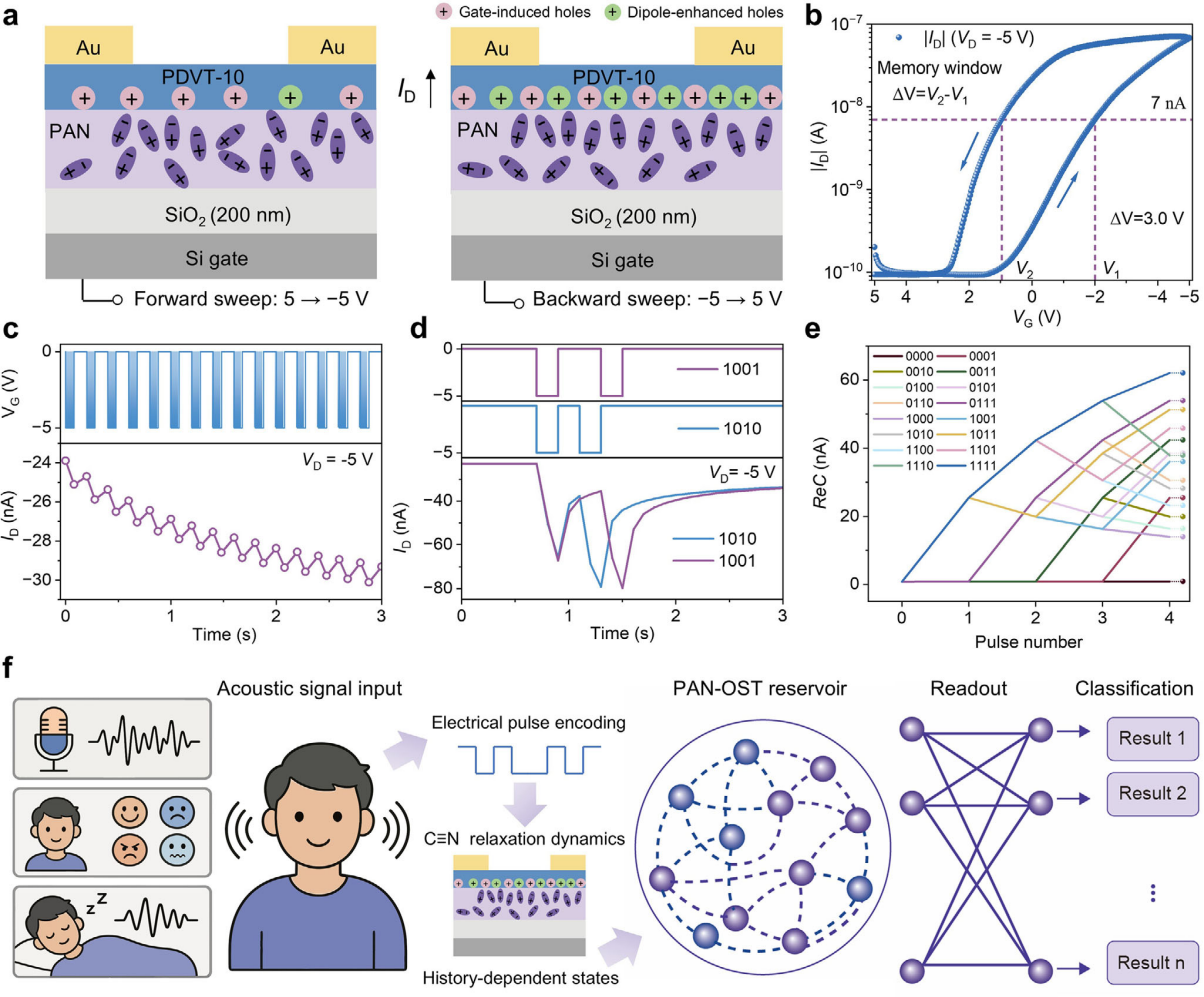
Acoustic RC based on PAN‐OSTs. (a) Interfacial polarization during gate voltage sweeps. (b) Hysteretic transfer characteristics. (c) Current state evolution under gate voltage pulses. (d) 4‐bit gate pulse encoding and current state responses. (e) Experimentally fitted current responses for 16 input states. (f) Acoustic RC framework.

Furthermore, an acoustic RC framework based on the PAN‐OST was constructed (Figure 1f). Acoustic signals were first converted into temporally encoded pulse sequences, which were then transformed into history‐dependent reservoir states by the experimentally derived nonlinear device dynamics. These states were subsequently processed by a trainable readout layer for classification.

### 2.2 Interfacial and Dipolar Characteristics of the PAN Interlayer

To establish the basis for the tunable ΔV, the PAN interlayer was systematically examined. UV–vis absorption spectra and ultraviolet photoelectron spectroscopy (UPS) analysis revealed favorable energy‐level alignment between PAN and PDVT‐10, consistent with efficient hole accumulation in the p‐type PDVT‐10 channel (Figure S2) [44]. Cross‐sectional transmission electron microscopy coupled with energy‐dispersive x‐ray spectroscopy (TEM‐EDS) elemental mapping revealed a clear vertical separation between the S‐rich PDVT‐10 (Figure S3) semiconductor layer and the N‐containing PAN interlayer, confirming the structural integrity of the device stack (Figure 2a and Figure S4) [45]. Scanning electron microscopy (SEM) and atomic force microscopy (AFM) analyses showed a smoother PDVT‐10 morphology on the PAN‐modified dielectric (Figures S5 and S6), while x‐ray diffraction (XRD) analysis showed nearly unchanged diffraction features compared with PDVT‐10 on bare SiO_2_, indicating improved interfacial quality between the dielectric and semiconductor without substantially perturbing semiconductor crystallinity (Figure S7). The control transistor without PAN exhibited inferior electrical characteristics (Figure S8) and no evident ΔV (Figure S9), confirming the essential role of the PAN interlayer in the observed hysteretic response. Interestingly, the hysteresis behavior changed markedly with PAN concentration. At 0.5 and 1 mg mL^−1^, AFM revealed discontinuous PAN coverage, and the devices showed clockwise hysteresis without an effective ΔV. At 2 and 4 mg mL^−1^, pronounced counterclockwise ΔV were observed. However, at 6 and 8 mg mL^−1^, the ΔV was strongly suppressed. The magnitude of ΔV also changed with the annealing time (15 or 30 min) (Figure S10). Therefore, the 2–5 mg mL^−1^ range was selected for further investigation of the tunable ΔV.

**FIGURE 2:**
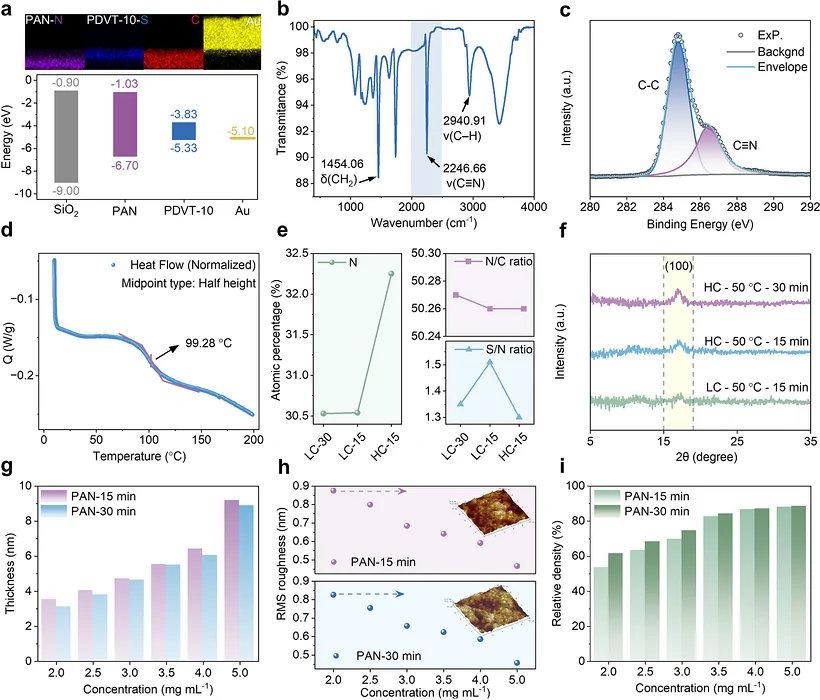
Interfacial characterization and structural evolution of PAN interlayers. (a) Energy‐level diagram and cross‐sectional TEM image and EDS elemental maps of the device stack. (b) FTIR spectrum of PAN powder. (c) C 1s XPS spectrum of PAN powder. (d) DSC thermogram of PAN powder. (e) XPS‐derived N atomic content, N/C ratio, and S/N ratio of the LC‐15, LC‐30, and HC‐15 PAN films. (f) XRD patterns of the LC‐15, HC‐15, and HC‐30 PAN films. LC and HC denote low and high PAN concentrations, respectively, whereas 15 and 30 denote annealing durations of 15 and 30 min, respectively. (g–i) Thickness, RMS roughness, and relative density of the PAN interlayers as functions of PAN concentration. All PAN films shown here were annealed at 50°C.

The presence of C≡N groups in PAN was further confirmed by Fourier transform infrared spectroscopy (FTIR) and x‐ray photoelectron spectroscopy (XPS). The FTIR spectrum exhibited a characteristic C≡N stretching band at 2246.66 cm^−1^ (Figure 2b) [46], while the C 1s and N 1s XPS spectra revealed chemical states associated with C≡N groups (Figure 2c and Figure S11) [47]. Differential scanning calorimetry (DSC) revealed a glass transition temperature (*T*_g_) of approximately 99.28°C for PAN (Figure 2d), indicating limited segmental mobility at 50°C, which is well below *T*_g_ [48]. In this polar glassy network, the C≡N units are expected to act as field‐responsive dipoles, while the constrained chain environment slows their orientational polarization and relaxation. This coupling between strong C≡N dipoles and restricted segmental dynamics favors a slow dipolar response.

To clarify how the C≡N dipole population was coupled to chain packing constraints, the PAN dipolar network was examined as a function of PAN concentration and annealing time. The N content increased with PAN concentration, indicating a greater contribution from C≡N groups in the PAN interlayer. The nearly unchanged N/C ratio suggested that this variation mainly originated from increased PAN loading rather than changes in the chemical composition of the interlayer. Meanwhile, the low S/N ratio and its further decrease after prolonged annealing indicated a limited contribution from dimethyl sulfoxide (DMSO)‐related sulfur residues (Figure 2e and Figure S12). These compositional trends indicated that changes in the interlayer are primarily associated with the C≡N dipolar network of PAN rather than effects associated with residual solvent [49].

The XRD patterns showed a PAN (100) diffraction peak at 2θ ≈ 17° (Figure 2f) [50]. The peak intensity increased with PAN concentration and was further enhanced after prolonged annealing, consistent with increased PAN content and more ordered chain packing. This enhanced packing suggested a more constrained chain environment that could limit C≡N relaxation dynamics. AFM and x‐ray reflectivity (XRR) analyses further revealed the concentration‐dependent evolution of the PAN interlayer thickness, surface roughness, and relative density [51, 52, 53]. For both the 15 and 30 min annealing series, the PAN films thickness remained below 10 nm, confirming that PAN functioned as an ultrathin interfacial layer rather than a bulk dielectric layer (Figure 2g). Within this nanoscale interfacial regime, the PAN film maintained sub‐nanometer root‐mean‐square (RMS) roughness, and increasing PAN concentration further reduced the roughness, indicating improved morphological uniformity of the continuous PAN interlayer (Figure 2h and Figure S13). Meanwhile, XRR analysis showed that the relative density increased rapidly at low and intermediate concentrations and then gradually approached 88%, suggesting progressive densification of the PAN chain network (Figure 2i and Figure S14 andNote S1). Comparison of the series annealed for 15 and 30 min further indicated that prolonged annealing promoted denser chain packing within the PAN interlayer. These results reveal two concurrent changes in the PAN interlayer with increasing PAN concentration: an increasing population of C≡N dipolar units and tighter chain packing accompanied by interlayer densification.

### 2.3 Dipolar Competition in Memory Window Modulation

Figure 3 illustrates the nonmonotonic evolution of the ΔV, which can be interpreted by competition between increasing C≡N dipole population and constrained dipolar relaxation dynamics in the PAN interlayer. As shown in Figure 3a and Figure S15, ∆V initially increased with PAN concentration and subsequently decreased at higher concentrations, reaching a maximum of approximately 4 V. This trend was observed in both the 15 and 30 min annealing series, whereas devices annealed for 30 min exhibited smaller ∆V values than those annealed for 15 min. This reduction suggested that prolonged annealing modified the PAN chain environment and dipolar relaxation dynamics, thereby reducing the retention of the history‐dependent polarized state. The nearly eliminated ΔV after annealing at 100°C (Figure S16) further suggested that elevated thermal treatment altered the relaxation dynamics of the PAN interlayer. Given that 100°C is close to the *T*_g_ of PAN, annealing at this temperature may enhance polymer segmental mobility and accelerate the relaxation of the interfacial polarized state, thereby suppressing ΔV. To exclude the influence of transistor characteristics, control devices without the PAN interlayer and statistical analyses of PAN‐interlayer devices were further evaluated. Compared with the control transistors (Figure S17), the PAN‐interlayer devices maintained low leakage current (Figure S18) and favorable transistor characteristics, including a high on/off ratio (Figure S19a,b), an onset voltage closer to 0 V (Figure S19c) and a saturation mobility of up to 1.23 cm^2^ V*^−^* ^1^ s*^−^* ^1^ (Figure S19d and Tables S1–S3). These results indicate that the enhanced ΔV cannot be attributed to leakage artifacts or degraded transistor performance. These findings further support that the ΔV modulation is governed by the balance between dipolar responsiveness and relaxation dynamics: excessively accelerated relaxation reduced polarization retention, whereas overly constrained dipolar dynamics limited effective polarization modulation.

**FIGURE 3:**
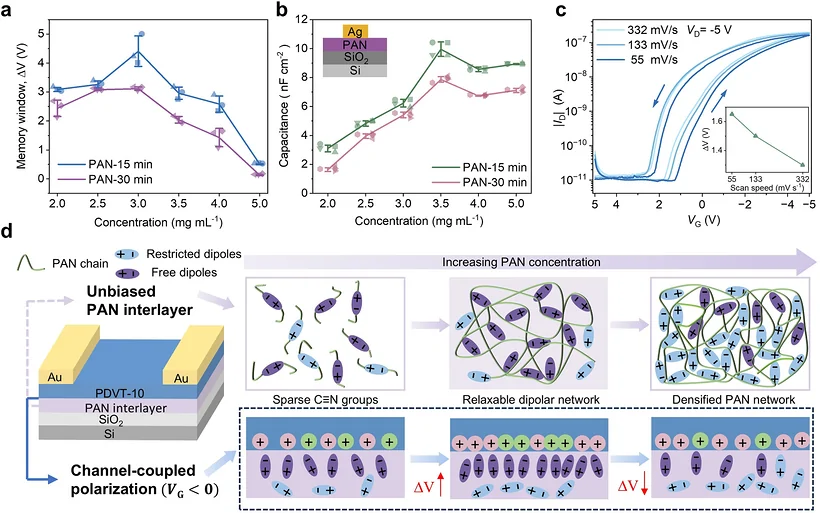
Concentration‐dependent ΔV characteristics and dipolar states of PAN interlayers. (a) ΔV as a function of PAN concentration after annealing at 50°C for 15 or 30 min. (b) Capacitance of SiO_2_/PAN dielectric stacks as a function of PAN concentration after annealing at 50°C for 15 or 30 min. (c) Sweep‐rate‐dependent transfer characteristics; inset: extracted ΔV as a function of sweep rate. (d) Schematic illustration of the concentration‐dependent dipolar states of the PAN interlayer, showing free and restricted C≡N dipoles with different degrees of dipolar relaxation freedom.

To further clarify the polarization and relaxation behavior of the PAN interlayer, the dielectric response of the SiO_2_/PAN stacks was examined. As shown in Figure 3b, the capacitance increased from low to intermediate PAN concentrations, reached a maximum at approximately 3.5 mg mL^−1^, and then decreased slightly while remaining relatively high at higher concentrations. The frequency‐dependent capacitance characteristics showed pronounced low‐frequency capacitance dispersion for both annealing series (Figure S20a,b), indicating the presence of slow polarization components in the PAN interlayer [54, 55]. Broadband dielectric spectroscopy (BDS) over 10^−2^–10^6^ Hz further revealed a decrease in capacitance with increasing frequency and a relaxation feature in the dielectric loss tangent (tan δ) (Figure S20c). These results indicate that polarization in the SiO_2_/PAN stack occurs over a finite relaxation time rather than instantaneously. This finite relaxation behavior provides the dielectric basis for residual polarization during gate voltage sweeping. However, the capacitance maximum did not coincide exactly with the maximum ∆V, indicating that the ΔV was not governed solely by the magnitude of polarization but also by the kinetics of C≡N dipolar polarization and relaxation. This kinetic contribution was further supported by sweep‐rate‐dependent transfer characteristics. As shown in Figure 3c, ∆V decreased as the gate voltage sweep rate increased from 55 to 332 mV s^−1^. The larger ΔV obtained at slower sweep rates indicates that C≡N dipolar polarization requires finite time to develop, while incomplete relaxation retains a history‐dependent interfacial state during the backward sweep, consistent with the dielectric relaxation [56]. In addition, a distinct ΔV was maintained over 50 consecutive transfer sweeps, with the forward and reverse *V*_G_ values remaining clearly separated throughout the cycling test (Figure S21), demonstrating reproducible history‐dependent current modulation under repeated operation.

Based on these results, the concentration‐dependent dipolar states of the PAN interlayer were summarized in Figure 3d. In the schematic, the two colors distinguish free and restricted C≡N dipoles, reflecting their different degrees of reorientation freedom. At low PAN concentrations, the limited C≡N dipole population resulted in weak residual polarization, leading to a small ΔV. At intermediate concentrations, the increased C≡N dipole population formed an effective dipolar network while retaining sufficient reorientation freedom, enabling stronger residual polarization and the largest ∆V. At high PAN concentrations, although a larger population of C≡N dipolar units was present, enhanced chain packing and interlayer densification restricted dipolar reorientation and suppressed the dynamic polarization response, thereby reducing the ΔV. Therefore, the nonmonotonic modulation of the ΔV is attributed to the competition between the increasing population of interfacial C≡N dipoles and the progressively constrained dipolar dynamics within the PAN interlayer. Considering both ΔV and transistor characteristics, the devices prepared with 3 mg mL^−1^ PAN and annealed at 50°C for 15 min were used for subsequent synaptic and reservoir computing measurements.

### 2.4 Synaptic Plasticity in PAN‐OSTs

To establish the synaptic dynamics of the PAN‐OST, *V*_G_ pulses were applied to a representative device with a pronounced ΔV. As shown in Figure 4a,b, increasing either the gate pulse amplitude or pulse duration enhanced the EPSC. The corresponding response current (*ReC*) and memory current (*MeC*) followed the same trends (Note S2) [57], confirming spike‐voltage‐dependent plasticity (SVDP) and spike‐duration‐dependent plasticity (SDDP) in the PAN‐OST (Figure S22a,b). The device generated a clearly distinguishable EPSC at a gate pulse amplitude as low as −100 mV, with a signal‐to‐noise ratio (SNR) of 50.47 dB and an event energy of only 50 aJ (Figure 4c and Note S2) [22]. The device exhibited an ultralow event energy relative to those reported for other synaptic transistors (Table S4).

**FIGURE 4:**
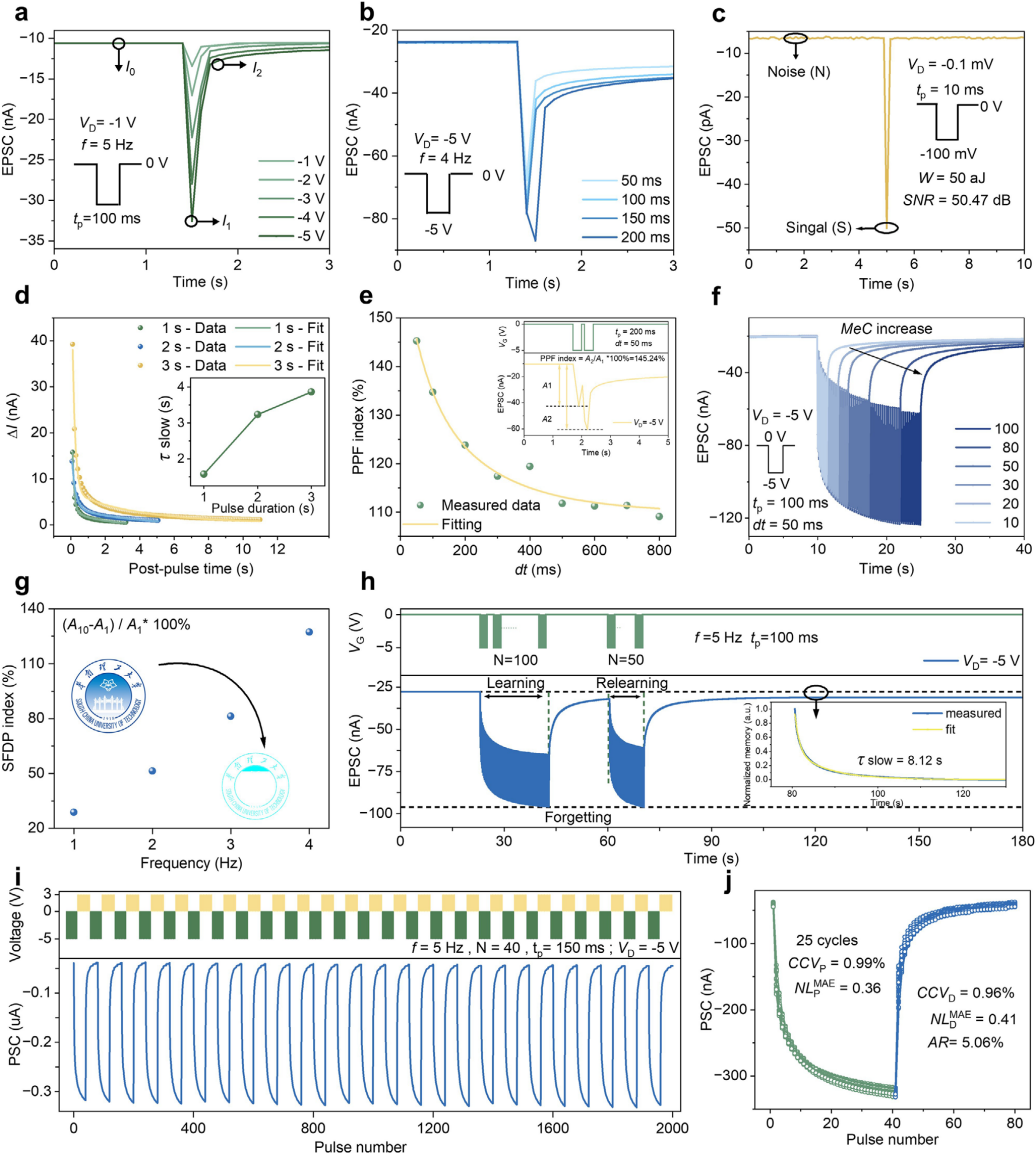
Dipolar relaxation and synaptic plasticity in PAN‐OSTs. (a) Pulse‐amplitude‐dependent EPSC responses measured with *V*_G_ varying from −1 to −5 V at *V*_D_ = −1 V, *f* = 5 Hz, and *t*_p_ = 100 ms. (b) Pulse‐duration‐dependent EPSC responses measured with t_p_ ranging from 50 to 200 ms at *V*_G_ = −5 V, *V*_D_ = −5 V, and *f* = 4 Hz. (c) Energy consumption and SNR. (d) Single pulse EPSC decay curves with biexponential fits after *V*_G_ = −5 V spikes with *t*_p_ = 1, 2, and 3 s; inset: extracted τ_slow_ values. (e) Biexponential fit to the PPF data; inset: PPF index, defined as *A*_2_/*A*_1_ × 100%. (f) Pulse‐number‐dependent EPSC measured with 10, 20, 30, 40, 50, 80 and 100 pulses at *V*_G_ = −5 V, dt = 50 ms, and *t*_p_ = 100 ms. (g) Frequency‐dependent SFDP index, defined as (*A*_10_−*A*_1_)/*A*_1_ × 100%. (h) Learning‐forgetting‐relearning behavior; inset: biexponential fit to the normalized memory decay. (i) Cyclic LTP/LTD behavior over 25 cycles of potentiation and depression. (j) Overlaid LTP/LTD curves over 25 cycles and the extracted weight update parameters, including $NL_{P}^{MAE}$, $NL_{D}^{MAE}$, *AR*, CCV_P_, and CCV_D_. *V*_D_ denotes the drain voltage, *t*_p_ denotes the pulse duration, *dt* denotes the interval between two consecutive gate pulses, and *f* denotes the pulse frequency.

To clarify the relaxation dynamics of the post spike state, single pulse EPSC decay was analyzed after gate voltage pulses with durations of 1, 2, and 3 s (Figure 4d). The EPSC decay curves were fitted using a biexponential relaxation model:

(1) $$\Delta I\;\left(t\right)=A_{\mathrm{fast}}e^{-t/\tau_{\mathrm{fast}}}+A_{\mathrm{slow}}e^{-t/\tau_{\mathrm{slow}}}+\Delta I_{\mathrm{res}}$$

where *A*_fast_ and *A*_slow_ are the amplitudes of the fast and slow relaxation components, τ_fast_ and τ_slow_ are the corresponding time constants, and Δ*I*_res_ represents the residual EPSC component [58]. Notably, τ_slow_ increased from 1.57 to 3.86 s as the pulse duration was extended from 1 to 3 s, indicating that prolonged gate stimulation generated a more persistent residual interfacial state (Table S5). This incomplete relaxation was further evidenced by PPF. At a pulse interval of 50 ms, the PPF index reached 145.24%, indicating that the state retained after the first spike enhanced the response to the second spike. The PPF data were further fitted using a biexponential decay function (Note S3), yielding two characteristic relaxation time constants of 66.59 and 224.79 ms, corresponding to the fast and slow decay components, respectively (Figure 4e and Figure S23) [59]. Together, these results support short‐term memory (STM) associated with C≡N dipolar relaxation.

As shown in Figure 4f, the EPSC response increased progressively as the number of gate pulses increased from 10 to 100, demonstrating spike‐number‐dependent plasticity (SNDP). Correspondingly, *ReC* increased from 81.75 to 103.15 nA, and *MeC* increased from 23.39 to 37.87 nA (Figure S22c). By comparison, the control transistor without PAN exhibited substantially weaker PPF and only limited cumulative modulation under consecutive gate pulses (Figure S24), highlighting the critical role of the PAN interlayer in establishing history‐dependent synaptic responses. The spike‐frequency‐dependent plasticity (SFDP) index increased with stimulation frequency, showing high‐pass‐like filtering behavior (Figure 4g and Figure S22d) [40]. The learning‐forgetting‐relearning behavior further highlighted the history dependence of the current state. As shown in Figure 4h, during the first learning stage, 100 gate pulses progressively increased the EPSC. After an 18 s forgetting interval, the EPSC remained above its initial level. During relearning, only 50 gate pulses were needed to recover a comparable EPSC level, indicating that the previous stimulation history enabled more efficient relearning. A biexponential fit to the forgetting process yielded a slow relaxation time constant of 8.12 s (Note S4), reflecting the gradual decay of the current state established during learning [30]. Even after an extended forgetting period of 180 s, the EPSC remained above its initial level, suggesting partial retention of the established current state. To visualize this history‐dependent evolution, the normalized EPSC was mapped onto the brightness of a horse image, illustrating the buildup, fading, and recovery (Figure S25).

To verify the repeatability of the current states, cyclic potentiation and depression measurements were performed. As shown in Figure 4i,j, the PAN‐OST maintained stable and reversible LTP/LTD behavior over 25 cycles. The weight‐update characteristics were quantitatively evaluated as detailed in Note S5, yielding mean absolute error (MAE)‐based nonlinearity (*NL*) values of $N\;L_{P}^{MAE}$= 0.359 ± 0.006 for potentiation and $N\;L_{D}^{MAE}$= 0.409 ± 0.006 for depression, together with a potentiation/depression asymmetry ratio (*AR*) of 0.051 ± 0.006. The average cycle‐to‐cycle variations were 0.99% for potentiation (*CCV*_P_) and 0.96% for depression (*CCV*_D_) [60]. These balanced potentiation and depression characteristics, together with the low cycle‐to‐cycle variations, indicate stable and reproducible updating of current states. The measured current states were further applied to the Modified National Institute of Standards and Technology (MNIST) handwritten‐digit recognition task, in which handwritten digits from 0 to 9 were classified into ten categories to evaluate their applicability to neuromorphic pattern recognition [61]. An accuracy of 90.82% was achieved (Figure S26). Overall, these results establish that PAN dipolar dynamics enable current states to be modulated, accumulated, relaxed, and repeatedly updated, thereby supporting subsequent RC.

### 2.5 PAN Reservoir for Spoken Digit Recognition

To exploit the PAN dynamics for acoustic temporal processing, a spoken digit recognition framework based on a PAN reservoir was developed. The Free Spoken Digit Dataset (FSDD), comprising 3000 audio samples of spoken digits from “0” to “9”, was used for this task. As illustrated in Figure 5a, each audio waveform was first converted into a 64 × 64 time‐frequency spectrogram (*T* ×*F*). At each time step, the corresponding 64‐dimensional spectral feature vector (*X*_n,t_) was encoded using the masks (*N*_m_ = 10). Each mask generated 15 pulse‐width‐encoded inputs (*p* = 15), which were sequentially processed using the PAN dynamics. The resulting current responses were sampled at 35 virtual nodes for each mask (*K* = 35), yielding 350 reservoir states per time step. These reservoir states were then concatenated into feature vectors and classified using a ridge regression readout (*W*_out_).

**FIGURE 5:**
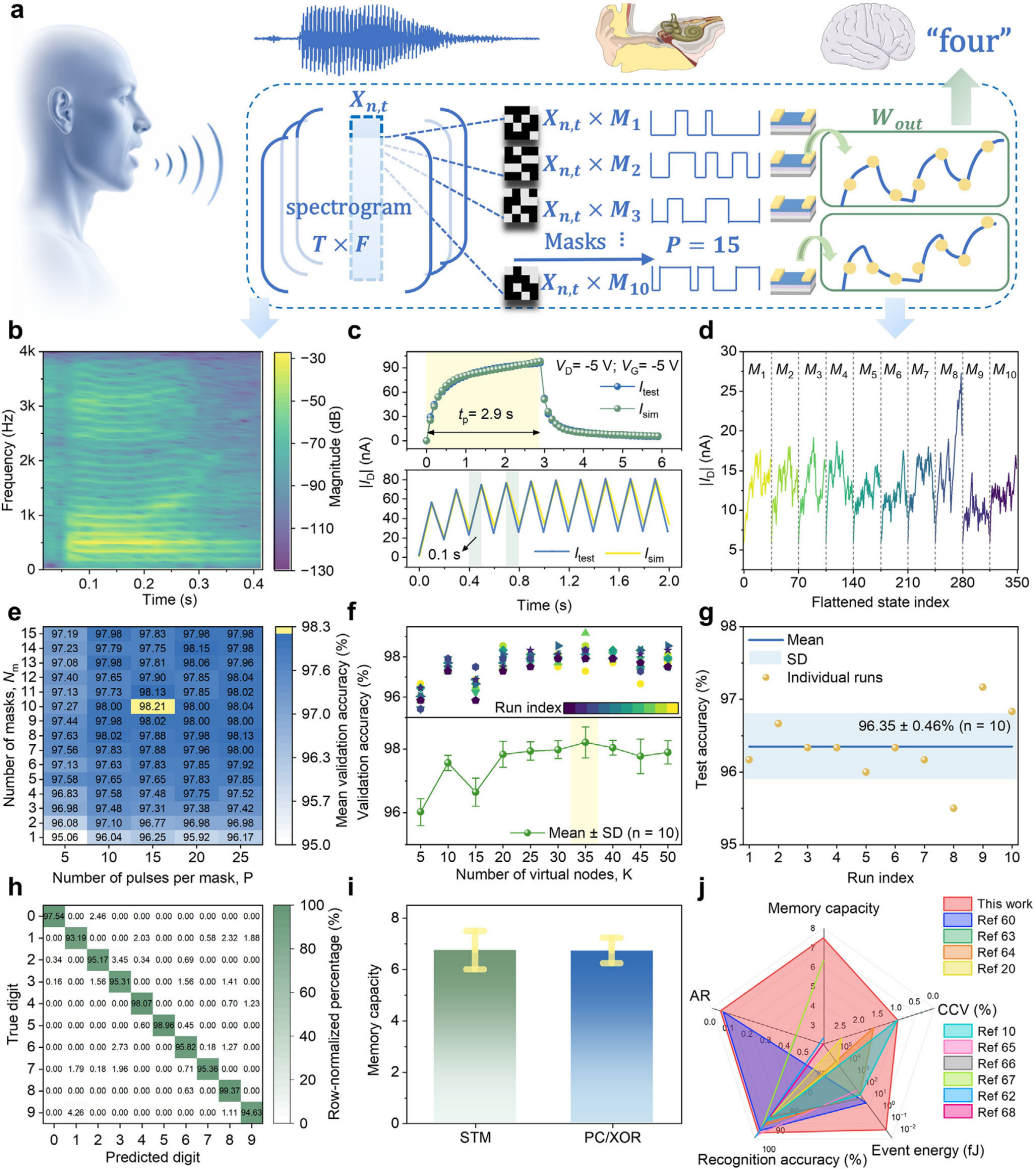
Spoken digit recognition based on PAN reservoir dynamics. (a) Workflow for spoken digit recognition. (b) Spectrogram of a representative spoken digit. (c) Measured and simulated current responses to single and successive pulses. (d) Flattened |*I*_D_| reservoir‐state sequence at the first time‐step, comprising 10 masks and 35 virtual nodes per mask (*N*_m_ = 10, *K* = 35). (e) Mean validation accuracy as a function of *N*_m_ and *P* with fixed *K* = 35 over ten independent runs. (f) Mean validation accuracy as a function of *K* with fixed *N*_m_ = 10 and *p* = 15 over ten independent runs. (g) Test accuracy with the final reservoir configuration (*K* = 35, *N*_m_ = 10, *p* = 15) over ten independent runs. (h) Pooled row‐normalized confusion matrix for digit classification over ten independent runs. (i) STM and PC/XOR capacities of the PAN reservoir. (j) Radar‐chart comparison of memory capacity, CCV, event energy, recognition accuracy, and AR between this work and representative reported neuromorphic devices.

A spectrogram of a representative spoken digit is shown in Figure 5b. To characterize the PAN dynamics for reservoir state generation, the rising current during pulse stimulation and the subsequent decay after pulse removal were extracted by fitting the single‐pulse response, yielding *f*_rise_ and *f*_down_, respectively (Figure 5c and Note S6). Without additional fitting parameters, these functions reproduced the measured current response under ten consecutive gate pulses, demonstrating that the single‐pulse‐derived dynamics described the current evolution of the PAN device under sequential pulse stimulation. The masked and pulse‐width‐encoded inputs were subsequently processed using these dynamics to generate reservoir states for the ridge regression readout. Accordingly, Figure 5d presents the 350 reservoir states generated at the first time‐step, while the complete reservoir‐state sequence over all 64 time‐steps is provided in Figure S27.

The reservoir parameters were optimized based on validation accuracy because *N*_m_, *P*, and *K* respectively govern the diversity of masked representations, temporal encoding length, and virtual‐node sampling resolution. For *N*_m_, too few masks provide insufficiently diverse mappings of the input, whereas increasing *N*_m_ generates a broader set of reservoir responses; however, further increasing *N*_m_ may produce highly correlated states with limited additional information. *P* determines the pulse‐width‐encoded temporal representation applied to the PAN dynamics, and variations in *P* therefore alter the temporal input pattern and the resulting reservoir states. For *K*, too few virtual nodes provide insufficient sampling of the transient device response, whereas increasing *K* captures more of its temporal evolution; when *K* becomes too large, neighboring sampled states may become highly correlated and mainly introduce redundancy. Consequently, the recognition accuracy depends on an appropriate combination of *N*_m_, *P*, and *K*, rather than simply increasing any individual parameter. To systematically evaluate these effects, *N*_m_ and *P* were first examined jointly with *K* = 35 fixed, with *N*_m_ varied from 1 to 15 and *P* varied over 5, 10, 15, 20, and 25. Each parameter combination was evaluated over ten independent runs. As shown in Figure 5e, the highest mean validation accuracy was obtained at *N*_m_ = 10 and *p* = 15, reaching 98.21% ± 0.48% (mean ± standard deviation (SD), n = 10). The corresponding SDs for all (*N*_m_, *P*) configurations are provided in Figure S28a. With *N*_m_ = 10 and *p* = 15 fixed, *K* was subsequently varied from 5 to 50 in increments of 5, and the highest mean validation accuracy was obtained at *K* = 35 (Figure 5f). The final reservoir configuration was therefore selected as *N*_m_ = 10 and *p* = 15, and *K* = 35. Using this fixed configuration, the mean test accuracy over ten independent runs was 96.35% ± 0.46%, with a maximum test accuracy of 97.17% (Figure 5g and Tables S6 and S7). The pooled raw‐count confusion matrix exhibited clearer diagonal dominance, indicating distinct class‐wise discrimination (Figure S28b), while the row‐normalized confusion matrix further showed robust recognition across all ten spoken digit classes (Figure 5h). The resulting recognition performance compared favorably with previously reported neuromorphic RC systems for spoken‐digit recognition (Table S8).

To complement the classification accuracy, STM and parity‐check/exclusive OR (PC/XOR) capacities of the PAN reservoir were evaluated to assess its temporal processing capability (Note S7) [62]. The corresponding capacities were 6.74 ± 0.75 for STM and 6.72 ± 0.49 for PC/XOR (Figure 5i and Table S9), indicating that the PAN dynamics supported both fading memory and nonlinear transformation, which were essential for RC. Strikingly, Figure 5j highlighted the highly competitive and well‐balanced performance of this work, which combined stable and symmetric weight updates with high recognition accuracy and memory capacity while maintaining an ultralow event energy [10, 20, 60, 62, 63, 64, 65, 66, 67, 68]. Together, these results demonstrate that the history‐dependent C≡N dipolar dynamics underpin efficient temporal information processing in the PAN reservoir.

### 2.6 Speech Emotion Recognition and Sleep Monitoring

In addition to linguistic content, acoustic signals can also convey affective states and physiological information related to sleep, making them valuable for human–machine interaction and health monitoring. Following validation on the spoken digit benchmark, the acoustic processing framework based on the PAN reservoir was extended to speech emotion recognition and snoring detection for sleep monitoring. As illustrated in Figure 6a, the two tasks underwent task‐specific acoustic preprocessing before pulse encoding. For speech emotion recognition, audio waveforms were converted into feature maps comprising mel‐frequency cepstral coefficients (MFCCs) and their first‐ and second‐order temporal derivatives (Δ and Δ^2^), yielding a 128 × 150 input map. For snoring detection, audio signals were converted into 64 × 62 mel‐spectrograms, which were subsequently binarized to form acoustic maps [69, 70]. These acoustic representations were then converted into pulse sequences and applied to the PAN reservoir. The experimentally fitted device dynamics transformed the pulse sequences into reservoir state trajectories. Finally, the resulting reservoir states were classified using task‐specific readouts: a linear readout for four‐class emotion recognition (Table S10) and a 992 × 2 readout based on the measured set and reset current updates for binary classification of snoring and non‐snoring sounds (Table S11 and Note S8) [6].

**FIGURE 6:**
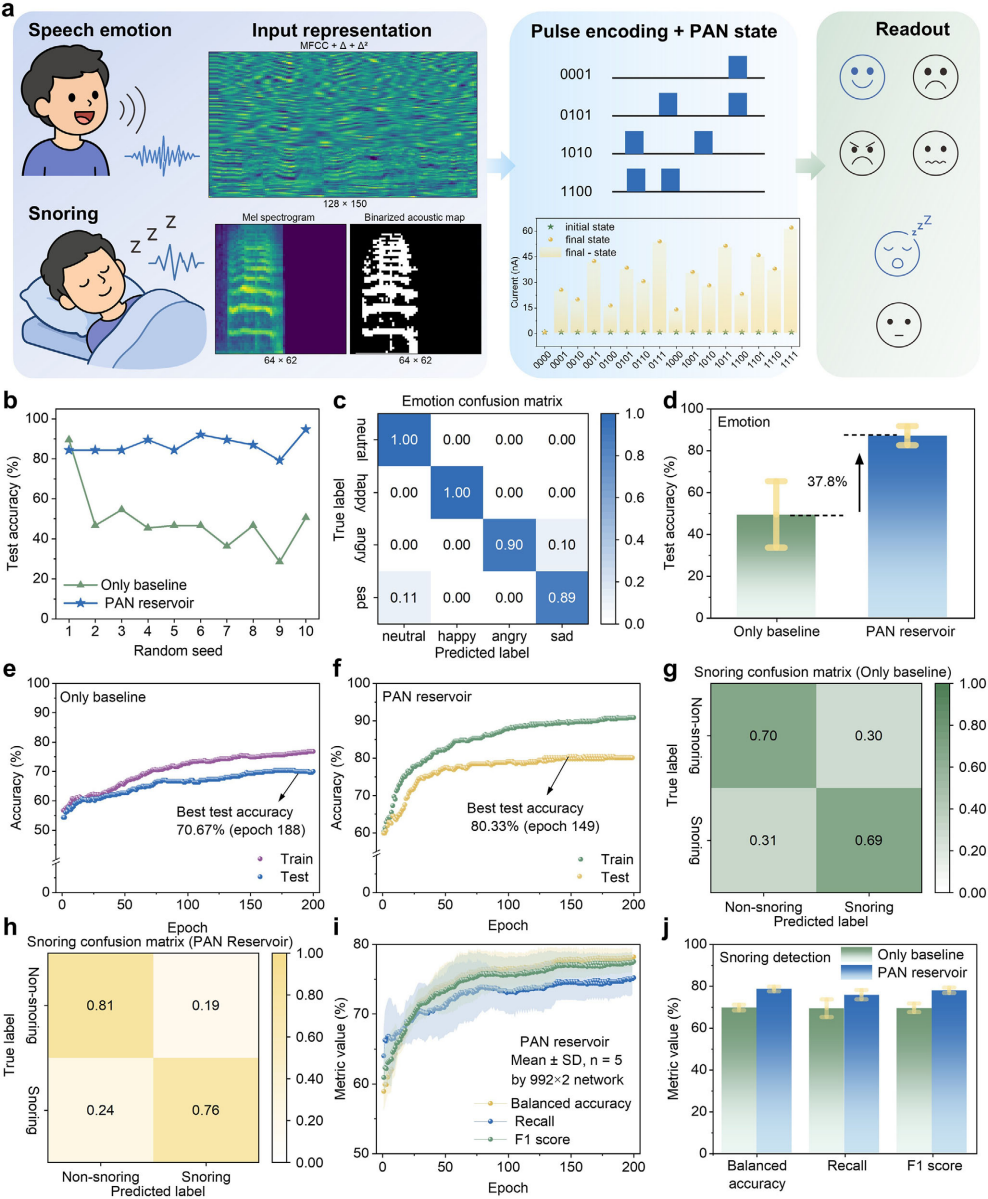
Speech emotion recognition and snoring detection based on PAN reservoir. (a) Workflow for speech emotion recognition and snoring detection, including task‐specific acoustic preprocessing, pulse encoding, PAN reservoir state generation, and readout classification. (b) Test accuracies for speech emotion recognition across ten random seeds. (c) Confusion matrix corresponding to the best‐performing random seed for speech emotion recognition. (d) Comparison of the mean test accuracies obtained with PAN reservoir and only baseline for speech emotion recognition. (e,f) Training and test accuracy curves for snoring detection with only baseline and PAN reservoir over 200 training epochs. (g,h) Row‐normalized pooled confusion matrices for only baseline and PAN reservoir, respectively, based on five independent runs at the corresponding best‐test epochs. (i) Evolution of balanced accuracy, recall, and F1 score for the PAN reservoir, shown as mean ± SD across five independent runs. (j) Comparison of balanced accuracy, recall, and F1 score between only baseline and PAN reservoir at the seed‐wise best‐test epochs; mean ± SD (n = 5).

For speech emotion recognition, the framework incorporating the PAN reservoir consistently outperformed the MFCC‐only baseline across ten random seeds (Figure 6b). The maximum test accuracy reached 94.8%, while the largest seed‐wise improvement was 53.2 percentage points. The confusion matrix corresponding to the best‐performing seed showed clear discrimination among the neutral, happy, angry, and sad classes (Figure 6c). The mean test accuracy increased from 49.2% ± 16.0% to 87.0% ± 4.6%, corresponding to an average improvement of 37.8 percentage points (Figure 6d). For snoring detection, the training and test accuracies increased over 200 training epochs for both the baseline and PAN reservoir, with the PAN reservoir exhibiting consistently higher test performance (Figure 6e,f). The baseline reached a best test accuracy of 70.67% at epoch 188, whereas the PAN reservoir achieved 80.33% at epoch 149 (Table S12). The row‐normalized confusion matrices, pooled across five independent runs (Table S13), further demonstrated improved discrimination between snoring and non‐snoring samples with the PAN reservoir compared with the baseline (Figure 6g,h). Consistently, the pooled confusion matrix counts showed fewer false‐positive (FP) and false‐negative (FN) predictions after PAN‐reservoir processing (Figure S29). To further evaluate the snoring‐detection performance, balanced accuracy was used to assess the overall discrimination between snoring and non‐snoring samples by accounting for both classes, while recall was used to evaluate the sensitivity to snoring events. The harmonic mean of precision and recall (F1 score) was further used to evaluate the balance between correctly detecting snoring events and avoiding false alarms. As shown in Figure 6i, the balanced accuracy, recall, and F1 score of the PAN reservoir increased progressively with training and gradually approached stable levels over 200 epochs. In comparison, the corresponding metrics for the baseline remained at lower levels (Figure S30), demonstrating the superior snoring detection performance of the PAN reservoir across five independent runs. At the best‐test epoch for each seed, balanced accuracy increased from 69.60% ± 1.36% for the baseline to 78.53% ± 1.17% for the PAN reservoir. Recall increased from 69.33% ± 4.24% to 75.73% ± 2.24%, while the F1 score increased from 69.47% ± 2.03% to 77.91% ± 1.32% (Figure 6j and Tables S14 and S15). These results corresponded to improvements of 8.93, 6.40, and 8.44 percentage points in balanced accuracy, recall, and F1 score, respectively. Together with the spoken digit recognition results, these findings demonstrate that the PAN dynamics convert diverse acoustic inputs into distinguishable reservoir states, supporting a unified PAN reservoir framework for spoken digit recognition, speech emotion recognition, and snoring detection for sleep monitoring.

## 3 Conclusion

In summary, this work elucidated the interfacial dipolar competition governing ΔV modulation in PAN‐OSTs. The ultrathin PAN layer formed a C≡N‐rich dipolar interface, where finite‐time polarization and relaxation imparted history dependence to channel modulation. With increasing PAN concentration, stronger dipolar polarization competed with increasingly constrained relaxation dynamics, resulting in nonmonotonic evolution of ΔV. The multiscale dynamics of the interfacial dipolar state gave rise to synaptic responses including SVDP, SDDP, SNDP, SFDP, and PPF (145.24%) to learning‐forgetting‐relearning behavior and LTP/LTD (AR = 5.06%, CCVs < 1%). Notably, the PAN synaptic transistor operated with an ultralow event energy of only 50 aJ while maintaining an exceptionally high signal‐to‐noise ratio of 50.47 dB. These PAN dynamics were then incorporated into the reservoir framework to map acoustic inputs onto separable representations, supporting spoken digit recognition (97.17%), speech emotion recognition (94.8%), and snoring detection (80.33%). Taken together, these results demonstrated that tuning the interfacial dynamics of C≡N dipoles enabled history‐dependent current modulation and fading memory for acoustic RC. This work established interlayer dipolar engineering as a viable route to harness interfacial polarization dynamics for temporal information processing in organic neuromorphic devices.

## 4 Method

### 4.1 Materials

PAN (average molecular weight ≈150 000), DMSO (AR grade, >99%), and chlorobenzene (CB, HPLC grade, 99.9%) were purchased from Sigma–Aldrich. PDVT‐10 (*M*w > 50 kDa, PDI < 3.0) was purchased from 1‐Material. All chemicals were used as received.

### 4.2 Device Fabrication

PAN was dissolved in DMSO at different concentrations and stirred at 60°C for 12 h to prepare the PAN solutions. PDVT‐10 was dissolved in chlorobenzene at a concentration of 5 mg mL^−1^ and stirred at 80°C for 12 h. Before use, all solutions were filtered through 0.22 um PTFE syringe filters.

Heavily doped Si (Si^++^)/SiO_2_ (200 nm) substrates were sequentially ultrasonicated in isopropanol, deionized water, acetone, and isopropanol for 10 min each. The cleaned substrates were dried at 80°C and plasma‐treated at 50 W for 5 min before film deposition. The PAN interlayer was spin‐coated onto the Si/SiO_2_ substrates at 2000 rpm for 60 s, followed by thermal annealing at 50°C for 15 or 30 min. Subsequently, the PDVT‐10 semiconductor layer was spin‐coated at 3000 rpm for 60 s, followed by annealing at 100°C for 30 min. Finally, 80 nm‐thick Au source and drain electrodes were deposited by thermal evaporation through a shadow mask. The channel width and length were 1100 and 200 um, respectively, as shown in Figure S31.

### 4.3 Characterization and Electrical Measurements

The electrical and synaptic characteristics of the PAN‐OSTs were measured in ambient air using a B1500A semiconductor parameter analyzer (Keysight Technologies), with gate voltage pulses supplied by a DG4202 pulse generator (RIGOL Technologies). XRD and XRR measurements were performed using an x‐ray diffractometer (Panalytical) with Cu Kα radiation (λ = 1.5405 Å). XPS and UPS spectra were recorded using a Nexsa spectrometer (Thermo Fisher Scientific, USA). Cross‐sectional TEM‐EDS elemental mapping was performed using a Talos F200X G2 transmission electron microscope (Thermo Fisher Scientific, USA). SEM images were acquired using an SU8220 scanning electron microscope (Hitachi, Japan). UV–vis absorption spectra were recorded using an Evolution 350 UV–vis spectrophotometer (Thermo Fisher Scientific, USA). AFM images were acquired using a Dimension Edge atomic force microscope (Bruker, Germany). FTIR spectra were recorded using a Nicolet iS20 spectrometer (Thermo Fisher Scientific, USA). DSC measurements were performed using a DSC 25 differential scanning calorimeter (TA Instruments, USA). BDS measurements were performed using an Alpha‐A broadband dielectric spectrometer (Novocontrol Technologies, Germany).

### 4.4 Acoustic Datasets and Computational Methods

The acoustic recognition tasks were implemented through numerical simulations within a common PAN RC framework. Spoken digit recognition was performed using FSDD, speech emotion recognition using the audio speech subset of RAVDESS, and snoring detection using the Kaggle snoring dataset. The acoustic signals were converted into task specific feature matrices, normalized, and encoded into pulse sequences. These pulse sequences were then transformed into history dependent reservoir states using the experimentally fitted current rise and decay dynamics of the PAN‐OSTs. Virtual nodes and time gating were used to expand the reservoir dimensionality.

For spoken digit and speech emotion recognition, the generated reservoir states were classified using ridge regression readouts with task‐dependent regularization. For snoring detection, a 992 × 2 readout based on the measured set and reset current updates was used. Detailed acoustic preprocessing procedures, reservoir configurations, readout methods, and evaluation procedures were provided in the Supporting Information.

## Author Contributions

**Yutian Liu**: writing – original draft, writing – review and editing, conceptualization, methodology, software, data curation, investigation, validation, formal analysis, visualization. **Jiale Huang**: software, validation, investigation. **Haoran Fang**: software, validation, methodology. **Jichen Zhao**: data curation, methodology, validation, visualization. **Fan Ye**: formal analysis, validation. **Bo Huang**: methodology, conceptualization. **Ruibin Liang**: validation, investigation. **Jintao Xu**: formal analysis, data curation. **Fanqi Wang**: formal analysis, methodology. **Mingjun Huang**: formal analysis, validation. **Zhiming Lin**: formal analysis, validation. **Shuguang Zhang**: validation, supervision, project administration, writing – review and editing, resources. **Linfeng Lan**: conceptualization, supervision, formal analysis, funding acquisition, project administration, resources, writing – review and editing. **Junbiao Peng**: resources, project administration.

## Ethics Statement

The datasets used in this work are obtained from publicly available sources, including MNIST, FSDD, RAVDESS, and Kaggle snoring dataset. All experiments in this work do not involve related ethics problems.

## Conflicts of Interest

The authors declare no conflicts of interest.

## Supporting information




**Supporting File**: advs78389‐sup‐0001‐SuppMat.docx.

## Data Availability

The data that support the findings of this study are available from the corresponding author upon reasonable request.
