# Supplementary material for: Interfacial Polarization Dynamics of Nitrile Dipoles in Organic Synaptic Transistors for Acoustic Reservoir Computing
Source: Adv Sci (Weinh). 2026 Oct 20:e78389. Online ahead of print. doi: 10.1002/advs.78389 (PMC13683882; doi:10.1002/advs.78389)
Supplement: Supplementary file 1 [file ADVS-9999-e78389-s001.docx]

Supporting Information

Interfacial Polarization Dynamics of Nitrile Dipoles in Organic Synaptic Transistors for Acoustic Reservoir Computing

*Yutian Liu^1^, Jiale Huang^1^, Haoran Fang^1^, Jichen Zhao^1^, Fan Ye^2^, Bo Huang**^3^, Ruibin Liang^1^, Jintao Xu^1^, Fanqi Wang^1^, Mingjun Huang^2^,* *ZhimingLin^4^, Shuguang Zhang^1^*, Linfeng Lan^1^*, Junbiao Peng^1^*

**Contents**

**1. Supporting Figures**

| Figures S1-S31 |  |
| --- | --- |

**2. Supplementary Notes**

| Supplementary Note 1. Calculation of the relative density of PAN interlayers. | Eqs. 1 |
| --- | --- |
| Supplementary Note 2. Calculation of *ReC*, *MeC*, SNR, and event energy. | Eqs. S2-6 |
| Supplementary Note 3. PPF decay fitting. | Eqs. S7-8 |
| Supplementary Note 4. Forgetting decay fitting. | Eqs. S9-10 |
| Supplementary Note 5. Weight-update parameter extraction. | Eqs. S11-24 |
| Supplementary Note 6. Spoken digit recognition based on a PAN reservoir. | Eqs. S25-35 |
| Supplementary Note 7. Memory capacity evaluation. | Eqs. S36-39 |
| Supplementary Note 8. Snoring detection based on a PAN reservoir. | Eqs. S40-44 |

**3. Supporting Tables**

| Summary *I*_on_, *I*_off_, on/off ratio and maximum \|*I*_G_\| of PAN-OSTs under different PAN concentrations and annealing times. | Table S1 |
| --- | --- |
| Mean values and standard deviations of *I*_on_, *I*_off_, *I*_on_ / *I*_off_, and maximum \|*I*_G_\| for PAN-OSTs at different PAN concentrations and annealing times. | Table S2 |
| *V*_on_ and *μ*_sat_ of PAN- OSTs at different PAN concentrations and annealing times. | Table S3 |
| Comparison of event energy consumption in reported synaptic transistor devices. | Table S4 |
| Biexponential fitting parameters for EPSC decay under different pulse widths. | Table S5 |
| Hyperparameters used for PAN reservoir based spoken digit recognition. | Table S6 |
| Test accuracy of spoken digit recognition over ten independent runs. | Table S7 |
| Comparison of the spoken digit recognition performance based on various neuromorphic devices. | Table S8 |
| Memory capacity results of the PAN reservoir. | Table S9 |
| Parameters and performance summary for speech emotion recognition. | Table S10 |
| Parameters for PAN reservoir assisted snoring detection. | Table S11 |
| Confusion-matrix outcomes at the best test epoch across five seeds for only baseline and PAN reservoir. | Table S12 |
| Pooled confusion-matrix counts and row-normalized values for snoring detection with only baseline and PAN reservoir. | Table S13 |
| Classification metrics at the best-test epoch across five independent runs for only baseline and the PAN reservoir. | Table S14 |
| Performance comparison of snoring detection between only baseline and PAN reservoir. | Table S15 |

**4. References**

**Figure S1.** a) Chemical structure of PAN. b) Polarity of the C≡N group in the PAN monomer, with C^δ⁺^ and N^δ⁻^.

**Figure S2.** a) Tauc plot of PAN. b) UPS secondary electron cutoff spectrum of PAN. c) UPS valence band onset spectrum of PAN. d) Tauc plot of PDVT-10. e) UPS secondary electron cutoff spectrum of PDVT-10. f) UPS valence-band onset spectrum of PDVT-10. g, h) Energy-level diagrams of PAN and PDVT-10, respectively.

**Figure S3.** Chemical structure of PDVT-10.

**Figure S4.** Cross-sectional HAADF-STEM image and corresponding EDS elemental maps of the PAN-interlayer PDVT-10 transistor, showing the spatial distributions of Au, C, N, and S.

**Figure S5.** a) SEM image of the PDVT-10 film spin-coated on bare SiO_2_. b) SEM image of the PDVT-10 film spin-coated on the PAN-modified SiO_2_ substrate.

**Figure S6.** a) AFM height image of the PDVT-10 film spin-coated on bare SiO_2_. b) AFM height image of the PDVT-10 film spin-coated on PAN-modified SiO_2_.

**Figure S7.** XRD patterns of PDVT-10 and PAN/PDVT-10 films.

**Figure S8.** Optimization of electrical characteristics of SiO_2_/PDVT-10 control transistors. a) Dependence of |*I*_on_|, |*I*_off_|, and |*I*_on_|/|*I*_off_| on PDVT-10 concentration. b) Dependence of mobility and $V_{on}$ on PDVT-10 concentration. c) Dependence of |*I*_on_|, |*I*_off_|, and |*I*_on_|/|*I*_off_| on spin-coating speed. d) Dependence of mobility and *V*_on_ on spin-coating speed.

**Figure S9.** Transfer characteristics of the optimized SiO_2_/PDVT-10 control transistor without a PAN interlayer measured at *V*_D_ = -15 V.

**Figure S10.** Concentration-dependent transfer characteristics and surface morphology of PAN/PDVT-10 transistors. a) Transfer characteristics of devices with 0.5 and 1 mg mL^–1^ PAN interlayers. b) Corresponding AFM images of the 0.5 and 1 mg mL^–1^ PAN interlayers. c,d) Transfer characteristics of devices with 2 and 4 mg mL^–1^, and 6 and 8 mg mL^–1^ PAN interlayers, respectively. All PAN interlayers were annealed at 50 °C for 15 or 30 min. All transfer characteristics were measured at *V*_D_ = –15 V.

**Figure S11.** N 1s XPS spectrum of PAN powder, showing the characteristic C≡N signal.

**Figure S12.** N 1s, C 1s, and S 2p XPS spectra of PAN films annealed at 50 °C. a-c) low concentration, 15 min. d-f) low concentration, 30 min. g-i) high concentration, 15 min.

**Figure S13.** AFM height images of PAN films spin-coated from solutions of 2, 2.5, 3, 3.5, 4, and 5 mg mL^−1^ (left to right) and annealed at 50 °C for a-f) 15 min and g-l) 30 min.

**Figure S14.** Densities of PAN interlayers as a function of PAN concentration after annealing at 50 °C for 15 or 30 min.

**Figure S15.** Transfer characteristics of PDVT-10 transistors with PAN interlayers annealed at 50 °C under different conditions. a-c) 2.0, 2.5, and 3.0 mg mL^–1^, respectively, for 15 min. d-f) 2.0, 2.5, and 3.0 mg mL^−1^, respectively, for 30 min. g-i) 3.5, 4.0, and 5.0 mg mL^−1^, respectively, for 15 min. j-l) 3.5, 4.0, and 5.0 mg mL^−1^, respectively, for 30 min. Three independent devices are shown for each condition. The dashed line indicates |*I*_D_| = 7 nA for memory-window extraction.

**Figure S16.** Output and transfer characteristics of PAN/PDVT-10 transistors prepared with a 3 mg mL^−1^ PAN solution and annealed at 100 °C for a, b) 30 min and c, d) 15 min.

**Figure S17.** Electrical characteristics of three independent PDVT-10 transistors without a PAN interlayer. a) transfer characteristics at *V*_D_ = −5 V. b) corresponding gate leakage currents.

**Figure S18.** Gate leakage currents of PDVT-10 transistors with PAN interlayers annealed at 50 °C under different conditions. a-c) 2.0, 2.5, and 3.0 mg mL^−1^, respectively, for 15 min. d-f) 2.0, 2.5, and 3.0 mg mL^−1^, respectively, for 30 min. g-i) 3.5, 4.0, and 5.0 mg mL^−1^, respectively, for 15 min. j-l) 3.5, 4.0, and 5.0 mg mL^−1^, respectively, for 30 min. Three independent devices are shown for each condition.

**Figure S19.** Electrical parameter statistics of PDVT-10 transistors with PAN interlayers prepared from solutions of different concentrations and annealed at 50 °C. a, b) |*I*_on_|, |*I*_off_|, and |*I*_on_|/|*I*_off_| after 15 and 30 min, respectively. c) *V*_on_ and d) *μ*_sat_ after 15 and 30 min.

**Figure S20.**Capacitance-frequency characteristics of SiO_2_/PAN dielectric stacks with PAN layers spin-coated from 2, 2.5, 3, 3.5, 4, and 5 mg mL^−1^ and annealed at 50 °C for a) 15 min b) 30 min. c) Frequency dependence of capacitance and dielectric loss tangent of a SiO_2_/PAN dielectric stack.

**Figure S21.** Cycling characteristics of a PAN/PDVT-10 transistor over 50 consecutive gate-voltage sweeps. a) transfer curves at *V*_D_ = –5 V. b) forward- and reverse-sweep *V*_G_ values at |*I*_D_|= 7 nA and the corresponding memory window.

**Figure S22.** *ReC* and *MeC* as a function of a) gate-pulse amplitude, b) pulse width, and c) pulse number. d) EPSC responses to repeated gate pulses at different frequencies.

**Figure S23.** PPF responses under different pulse intervals. a-h) Pulse intervals of 100, 200, 300, 400, 500, 600, 700, and 800 ms, respectively.

**Figure S24.** Synaptic responses of the control PDVT-10 transistor without the PAN interlayer. a) PPF and b) response to 25 consecutive gate pulses (*V*_G_ = –5 V, *t*_p_ = 200 ms, *f* = 4 Hz).

**Figure S25.** Simulated horse images illustrating the learning-forgetting-relearning behavior of the PAN/PDVT-10 synaptic transistor. The horse image was learned after 100 pulses within 18 s, faded after 18 s, relearned after 50 pulses within 9 s, and gradually forgotten over 120 s.

**Figure S26.** a) Three-layer neural network for MNIST digit recognition with 28 × 28 inputs and 10 output classes. b) Recognition accuracy using PAN/PDVT-10 synaptic transistors.

**Figure S27.** Complete |*I*_D_| reservoir-state sequence over 64 time-steps.

**Figure S28.** a) Sample SD of the validation accuracy as a function of *N*_m_ and *P* with fixed *K* = 35 over ten independent runs. b) Pooled raw-count confusion matrix of digit-classification outcomes across ten independent runs.

**Figure S29.** Pooled confusion matrices for snoring detection across five independent runs. a) baseline only. b) PAN reservoir. Rows and columns indicate true and predicted labels, respectively, with snoring as the positive class. TN, FP, FN, and TP denote true negatives, false positives, false negatives, and true positives, respectively.

**Figure S30.** Balanced accuracy, recall, and F1 score for baseline-only snoring detection over 200 training epochs. Solid lines and shaded regions indicate the mean and ±1 standard deviation across five independent runs, respectively.

**Figure S31.** Schematic illustration of the fabrication process of the PAN/PDVT-10 synaptic transistor.

**Supplementary Note 1. Calculation of the relative density of PAN interlayers.**

The relative density of the PAN interlayers was calculated from the film density obtained by X-ray reflectivity (XRR) fitting according to

$\rho_{\mathrm{relative}}=\frac{\rho_{\mathrm{film}}}{\rho_{\mathrm{theoretical}}}\times100\%$	(1)

where $\rho_{\mathrm{film}}$ is the PAN film density obtained from XRR fitting and $\rho_{\mathrm{theoretical}}$ is the theoretical crystalline density of PAN. A value of 1.2 g cm^–3^ was adopted for $\rho_{\mathrm{theoretical}}$, based on the reported calculated density of the PAN crystal unit cell (1.199 g cm^–3^) ^[1]^.

**Supplementary Note 2. Calculation of *ReC*, *MeC*, *SNR*, and event energy.**

The response current (*ReC*) and memory current (*MeC*) were calculated from the EPSC response as: ^[2-3]^

$ReC = |I_{1}-I_{0}|$	(2)

$MeC = |I_{2}-I_{0}|$	(3)

where $I_{0}$ is the baseline current before the gate-voltage spike, $I_{1}$ is the peak current during the EPSC response, and $I_{2}$ is the retained current measured at a fixed delay after removal of the gate-voltage spike. The absolute values are used to express *ReC* and *MeC* as positive current amplitudes.

The signal-to-noise ratio (*SNR*) was calculated as: ^[4-5]^

$A_{sig} = I_{sig}- I_{base}$	(4)

$SNR = 20\log_{10} ( \frac{|A_{sig}|}{\sigma_{base}} )$	(5)

where $I_{\mathrm{sig}}$is the EPSC signal current, $I_{\mathrm{base}}$ is the average baseline current before the gate-voltage spike, $A_{\mathrm{sig}}$ is the baseline-subtracted EPSC signal amplitude, and $\sigma_{\mathrm{base}}$ is the standard deviation of the baseline noise before the spike.

The event energy was estimated according to: ^[6]^

$E_{event} = \left| V_{D} \right|\times\left| I_{D} \right|\times t$	(6)

where $V_{D}$ is the drain voltage, $I_{D}$ is the drain current, and $t$ is the pulse duration.

**Supplementary Note 3. PPF decay fitting.**

PPF decay was fitted using a biexponential decay function: ^[7]^

$\mathrm{PPF}\left( \Delta t \right) = y_{0}+A_{1}exp(-\frac{\Delta t}{\tau_{1}})+A_{2}exp(-\frac{\Delta t}{\tau_{2}})$	(7)

where $\Delta t$ is the interval between two consecutive gate-voltage spikes, $y_{0}$ is the asymptotic PPF index at long pulse intervals, $A_{1}$ and $A_{2}$ are the amplitudes of the fast and slow decay components, respectively, and $\tau_{1}$ and $\tau_{2}$are the corresponding characteristic decay time constants.

For the PAN- OST, the fitted PPF decay is expressed as:

$\mathrm{PPF}\left( \Delta t \right) = 109.88+25exp(-\frac{\Delta t}{66.59})+30exp(-\frac{\Delta t}{224.79})$	(8)

where $\Delta t$ is given in milliseconds and the PPF index is expressed in percent. The extracted characteristic time constants are $\tau_{1}=66.59\mathrm{ms}$ and $\tau_{2}=224.79\mathrm{ms}$.

**Supplementary Note 4. Forgetting decay fitting.**

The forgetting decay after the 50-pulse relearning process was fitted using a biexponential relaxation function: ^[8]^

$M(t)=A_{\mathrm{fast}}exp(-\frac{t-t_{0}}{\tau_{\mathrm{fast}}})+A_{\mathrm{slow}}exp(-\frac{t-t_{0}}{\tau_{\mathrm{slow}}})$	(9)

where $M(t)$ is the normalized memory state after subtracting the residual current, $t_{0}$ is the starting time of the decay after the last gate-voltage spike, $A_{\mathrm{fast}}$ and $A_{\mathrm{slow}}$ are the amplitudes of the fast and slow relaxation components, respectively, and $\tau_{\mathrm{fast}}$and $\tau_{\mathrm{slow}}$are the corresponding characteristic relaxation time constants.

For the PAN- OST, the fitted forgetting decay is expressed as:

$M(t)=0.549exp(-\frac{t-80.605}{1.08})+0.380exp(-\frac{t-80.605}{8.12})$	(10)

where $t$ is in seconds. The extracted characteristic relaxation time constants are $\tau_{\mathrm{fast}}=1.08 s$and $\tau_{\mathrm{slow}}=8.12 s$.

**Supplementary Note 5. Weight-update parameter extraction.**

For the $i$-th potentiation and depression cycles, the conductance after the $n$-th programming pulse was denoted as $G_{P,i}(n)$and $G_{D,i}(n)$, respectively. Each cycle contained $N=40$ pulses, and 25 cycles were analyzed for both potentiation and depression.

The conductance values in each cycle were independently normalized to the range of 0-1. Because potentiation corresponds to an increase in conductance whereas depression corresponds to a decrease in conductance, the normalized depression sequence was converted into an increasing form for direct comparison of the two update profiles:

$G_{P,i}^{norm}(n)=\frac{G_{P,i}(n)-G_{P,i}^{min}}{G_{P,i}^{max}-G_{P,i}^{min}}$	(11)

$G_{D,i}^{norm}(n)=\frac{G_{D,i}(n)-G_{D,i}^{min}}{G_{D,i}^{max}-G_{D,i}^{min}}$	(12)

$G_{D,i}^{inc}(n)=1-G_{D,i}^{norm}(n)$	(13)

where $G_{P,i}^{\min}$, $G_{P,i}^{\max}$, $G_{D,i}^{\min}$, and $G_{D,i}^{\max}$are the minimum and maximum conductance values in the corresponding potentiation or depression cycle. $G_{P,i}^{\mathrm{norm}}(n)$and $G_{D,i}^{\mathrm{norm}}(n)$are the normalized potentiation and depression conductance sequences, respectively, and $G_{D,i}^{\mathrm{inc}}(n)$is the normalized depression sequence converted into an increasing form.

The ideal linear update profile was defined as:

$G_{ideal}\left( n \right)=\left( n-1 \right)/\left( N-1 \right)$	(14)

The nonlinearity was calculated as the mean absolute error (MAE) between the normalized conductance sequence and the ideal linear update profile: ^[9,10]^

$NL_{P,i}^{MAE}=\frac{1}{N}\sum_{n=1}^{N} \left| G_{P,i}^{norm}\left( n \right)-G_{ideal}\left( n \right) \right|$	(15)

$NL_{D,i}^{MAE}=\frac{1}{N}\sum_{n=1}^{N} \left| G_{D,i}^{inc}\left( n \right)-G_{ideal}\left( n \right) \right|$	(16)

The potentiation/depression asymmetry index, abbreviated as *AR*, was calculated as the mean absolute difference between the normalized potentiation sequence and the depression sequence converted into an increasing form: ^[9,10]^

${AR}_{i}=\frac{1}{N}\sum_{n=1}^{N} \left| G_{P,i}^{norm}\left( n \right)-G_{D,i}^{inc}\left( n \right) \right|$	(17)

A smaller *AR* value indicates lower potentiation/depression asymmetry and therefore more balanced conductance modulation.

The cycle-to-cycle variation (*CCV*) was calculated from the relative standard deviation of the normalized conductance values at the same pulse number among different cycles: ^[10-11]^

$CCV\left( n \right)=\frac{\sigma_{X}\left( n \right)}{\mid\mu_{X}\left( n \right)\mid+\varepsilon}\times100\%$	(18)

$CCV=\frac{1}{N}\sum_{n=1}^{N} CCV\left( n \right)$	(19)

where $X_{i}(n)$represents $G_{P,i}^{\mathrm{norm}}(n)$for potentiation and $G_{D,i}^{\mathrm{inc}}(n)$for depression. $\mu_{X}(n)$and $\sigma_{X}(n)$are the mean value and standard deviation of $X_{i}(n)$among 25 cycles at the $n$-th pulse, respectively. $\varepsilon$is a small constant used to avoid division by zero.

Using Eqs. (11)-(19), the extracted weight-update parameters were:

$NL_{P}^{MAE}=0.359\pm0.006$	(20)

$NL_{D}^{MAE}=0.409\pm0.006$	(21)

$AR=0.051\pm0.006$	(22)

$CCV_{P}=0.99\%$	(23)

$CCV_{D}=0.96\%$	(24)

**Supplementary Note 6. Spoken digit recognition based on a PAN reservoir.**

FSDD was used for spoken-digit recognition. The dataset contains 3000 mono WAV recordings of English spoken digits from 0 to 9, recorded by six speakers at a sampling rate of 8 kHz. ^[12]^ Each digit contains 300 recordings. In this work, the dataset was randomly split into training and test sets with an 80:20 ratio using a fixed random seed of 42. The split yielded 2400 training samples and 600 test samples. For hyperparameter optimization, the original training set was further divided into a training subset and a validation set and validation sets using a stratified 80:20 split with a fixed random seed of 2026, yielding 1920 sub-training samples and 480 validation samples. The test set was not used during hyperparameter optimization.

The audio samples were represented as $64\times64$ spectrogram images. During data loading, each spectrogram image was converted into a two-dimensional matrix. For multichannel images, the channel dimension was averaged. The pixel values were normalized by dividing by 255, yielding input matrices:

$\begin{matrix} & X\in\mathbb{R}^{N_{s}\times64\times64} & & \end{matrix}$	(25)

where $N_{s}$ is the number of audio samples.

For masked temporal encoding, each spectrogram matrix $X$was multiplied by random masks $M$, generating encoded pulse sequences: ^[13]^

$\begin{matrix} & U_{m,n,t,p}=\sum_{f} X_{n,t,f}M_{m,f,p} & & \end{matrix}$	(26)

where $X_{n,t,f}$ is the spectrogram value of the $n$-th sample at time index $t$ and frequency index $f$, $M_{m,f,p}$ is the random mask, $m$ is the mask index, and $p$ is the pulse index within each time step.

The encoded values were further converted into pulse-on durations:

$\begin{matrix} & T_{\mathrm{on}}=T_{\min}+(T_{\max}-T_{\min})\frac{U-U_{\min}}{U_{\max}-U_{\min}+\varepsilon} & & \end{matrix}$	(27)

$\begin{matrix} & T_{\mathrm{off}}=T_{\mathrm{cycle}}-T_{\mathrm{on}} & & \end{matrix}$	(28)

Where $T_{\max}=T_{\mathrm{cycle}}$, and $T_{\mathrm{cycle}}=1.0$ were used in the simulation.$\varepsilon$ is a small constant used to avoid division by zero.

The PAN reservoir dynamics were described by experimentally fitted current rise and decay functions:

$\begin{matrix} & f_{\mathrm{rise}}(x)=74.62e^{0.09333x}-74.62e^{-3.917x} & & \end{matrix}$	(29)

$f_{\mathrm{down}}(x)=11.41e^{-x/3.229}+38.22e^{-x/0.2113}+0.8565$	(30)

where $x$ is the elapsed time in seconds and the output current is given in nA. $f_{\mathrm{rise}}$ and $f_{\mathrm{down}}$ describe the experimentally fitted current rise and decay dynamics of the PAN-interlayer OST, respectively.

The PAN reservoir state was updated sequentially through pulse-on and pulse-off processes:

$I_{\mathrm{on}}=f_{\mathrm{rise}}\left[ f_{\mathrm{rise}}^{-1}(I_{\mathrm{prev}})+T_{\mathrm{on}} \right]$	(31)

$I_{\mathrm{next}}=f_{\mathrm{down}}\left[ f_{\mathrm{down}}^{-1}(I_{\mathrm{on}})+T_{\mathrm{off}} \right]$	(32)

where $I_{\mathrm{prev}}$ represents the output current state before the input pulse, $I_{\mathrm{on}}$ represents the current state after pulse-on stimulation, and $I_{\mathrm{next}}$ represents the relaxed current state after the pulse-off interval. $f_{\mathrm{rise}}^{-1}$ and $f_{\mathrm{down}}^{-1}$ represent the inverse mappings from current to the corresponding elapsed time.

Within each time-step, virtual node states were extracted from the PAN reservoir response. The temporal interval between adjacent virtual nodes was defined as: ^[14-15]^

$\begin{matrix} & \Theta_{T}=\frac{T_{\mathrm{cycle}}\times P}{K} & & \end{matrix}$	(33)

where $P$ is the number of pulses per time step and $K$ is the number of virtual nodes. The generated PAN reservoir states were flattened into feature vectors and fed to a linear readout with ridge regularization ($\lambda=100$) for spoken-digit classification.

The output weights of the linear readout were trained using ridge regression: ^[16]^

$\begin{matrix} & W_{\mathrm{out}}=argminW\left( {\parallel Y-XW\parallel}_{F}^{2} + \lambda{\parallel W\parallel}_{F}^{2} \right), & & \end{matrix}$	(34)

where $X$ is the PAN reservoir-state matrix, $Y$ is the target-label matrix, and $W_{\mathrm{out}}$ is the trained readout-weight matrix. The first term represents the prediction error, whereas the second term penalizes excessively large readout weights. The regularization coefficient $\lambda$ controls the strength of this penalty. A value of $\lambda=1\times{10}^{-5}$was used, providing weak regularization to improve numerical stability and reduce overfitting while retaining the fitting capability of the linear readout. Hyperparameter optimization and confusion matrix

The reservoir hyperparameters were evaluated using controlled validation analyses. With $K=35$ fixed, $N_{m}$ was varied from 1 to 15 and $P$ was varied over 5, 10, 15, 20, and 25. The highest mean validation accuracy was obtained at $N_{m}=10$ and $P=15$. Subsequently, with $N_{m}=10$ and $P=15$ fixed, $K$ was varied from 5 to 50 in increments of 5, yielding the highest mean validation accuracy at $K=35$. Each configuration was evaluated over ten independent runs using the same data split and fixed model settings. Accordingly, $K=35$, $N_{m}=10$, and $P=15$ were used as the final reservoir configuration.

After the hyperparameters were fixed, the final model was evaluated on the test set over ten independent runs. For each run, the ridge readout was trained using the complete 2400-sample training set and subsequently evaluated on the same 600-sample test set. The resulting test accuracy was $96.35\pm0.46\%$ (mean $\pm$ SD, $n=10$).

A pooled confusion matrix was further constructed from the classification outcomes across the ten independent runs. In total, 5781 of 6000 pooled classification outcomes were correctly classified, corresponding to an overall pooled accuracy of:

Accuracy = $\frac{5781}{6000}\times100\%=96.35\%$	(35)

The 6000 outcomes represent the pooled classification outcomes obtained from ten independent evaluations of the 600-sample test set.

**Supplementary Note 7. Memory capacity evaluation.**

Memory capacity was evaluated using random binary input sequences. For the STM task, the target output was defined as the delayed input: ^[17,18]^

$y_{\mathrm{STM}}(T,T_{\mathrm{delay}})=s_{\mathrm{in}}(T-T_{\mathrm{delay}})$	(36)

For the parity-check/XOR task, the target output was defined as: ^[17,18]^

$\begin{matrix} & y_{PC/XOR}(T,T_{\mathrm{delay}})=s_{\mathrm{in}}(T-T_{\mathrm{delay}})\oplus s_{\mathrm{in}}(T-T_{\mathrm{delay}}+1)\oplus\cdots\oplus s_{\mathrm{in}}(T) & & \end{matrix}$	(37)

where $s_{\mathrm{in}}(T)$ is the random binary input sequence and $\oplus$ denotes the XOR operation.

For each delay step, a linear readout was trained to predict the corresponding target output from the PAN reservoir states. The squared correlation coefficient was calculated as: ^[17,18]^

$\mathrm{Cor}^{2}(T_{\mathrm{delay}})=\frac{\mathrm{Cov}^{2}\left[ y_{\mathrm{target}}(T,T_{\mathrm{delay}}),y_{\mathrm{out}}(T) \right]}{\mathrm{Var}\left[ y_{\mathrm{target}}(T,T_{\mathrm{delay}}) \right]\mathrm{Var}\left[ y_{\mathrm{out}}(T) \right]}$	(38)

where $y_{\mathrm{target}}(T,T_{\mathrm{delay}})$ is the target output for a given delay time, and $y_{\mathrm{out}}(T)$ is the readout output.

The memory capacity was obtained by summing $\mathrm{Cor}^{2}$ over different delay steps: ^[19,20]^

$MC=\sum_{T_{\mathrm{delay}}=1}^{T_{\mathrm{delay},\max}} \mathrm{Cor}^{2}(T_{\mathrm{delay}})$	(39)

where $T_{\mathrm{delay},\max}=20$. The total sequence length was 60, with the first 40 time-steps used for training and the remaining 20 time-steps used for testing. Each task was repeated 30 times, and the error bars represent the standard deviation.

**Supplementary Note 8. Snoring detection based on a PAN reservoir.**

Snoring detection was performed as a binary classification task between snoring and non-snoring audio samples. Each audio sample was converted into a $64\times62$ mel-spectrogram and further binarized into an acoustic map. The binarized acoustic map was divided into 4-pixel input segments, resulting in:

$N_{\mathrm{input}}=\frac{64\times62}{4}=992$	(40)

input features for the readout.

The pulse-encoded inputs were processed by the PAN reservoir to generate reservoir-state responses. The resulting outputs were classified using a device-aware $992\times2$ readout based on experimentally measured set/reset conductance-update characteristics. The two output nodes corresponded to the snoring and non-snoring classes, respectively.

The classification performance was evaluated using balanced accuracy, recall, precision, and $F_{1}$ score, where the F1 score represents the harmonic mean of precision and recall. These metrics were used to evaluate both class-balanced discrimination and snoring-event detection capability.

Balanced accuracy was calculated as: ^[21,22]^

$\begin{matrix} & B\mathrm{alanced} \mathrm{accuracy}=\frac{1}{2}\left( \frac{TP}{TP+FN} + \frac{TN}{TN+FP} \right) & & \end{matrix}$	(41)

where $TP$, $TN$, $FP$, and $FN$ represent true positives, true negatives, false positives, and false negatives, respectively.

Recall was calculated as: ^[21,22]^

$\mathrm{Recall}=\frac{TP}{TP+FN}$	(42)

Precision was calculated as: ^[21,22]^

$\mathrm{Precision}=\frac{TP}{TP+FP}$	(43)

The $F_{1}$ score was calculated as: ^[21,22]^

$\begin{matrix} & F_{1} score=\frac{2\times\mathrm{Precision}\times\mathrm{Recall}}{\mathrm{Precision}+\mathrm{Recall}} & & \end{matrix}$	(44)

**Table S1. Summary of |*I*_on_|, |*I*_off_|, |*I*_on_|/|*I*_off_| and maximum *|I*_G_| of PAN-OSTs under different PAN concentrations and annealing times (n = 3)**

| PAN  Concentration  (mg/mL) | Annealing  (min) | Device | *\|I*_on_\| (A) | *\|I*_off_\| (A) | *\|I*_on_\|/*\|I*_off_\| | Max *\|I*_G_\| (A) |
| --- | --- | --- | --- | --- | --- | --- |
| — | — | 1 | 3.372 × 10^-7^ | 1.609 × 10^-7^ | 2.096 | 1.760 × 10^-8^ |
|  |  | 2 | 3.208 × 10^-7^ | 1.656 × 10^-7^ | 1.938 | 3.937 × 10^-8^ |
|  |  | 3 | 3.109 × 10^-7^ | 1.614 × 10^-7^ | 1.926 | 3.660 × 10^-8^ |
| 2.0 | 15 | 1 | 7.340 × 10^-8^ | 1.384 × 10^-10^ | 530.152 | 2.940 × 10^-10^ |
|  |  | 2 | 1.204 × 10^-7^ | 3.142 × 10^-10^ | 383.357 | 1.648 × 10^-9^ |
|  |  | 3 | 8.800 × 10^-8^ | 6.338 × 10^-11^ | 1388.380 | 3.605 × 10^-10^ |
|  | 30 | 1 | 9.971 × 10^-8^ | 1.307 × 10^-10^ | 762.876 | 5.085 × 10^-10^ |
|  |  | 2 | 6.090 × 10^-8^ | 1.880 × 10^-11^ | 3239.081 | 4.005 × 10^-10^ |
|  |  | 3 | 6.081 × 10^-8^ | 1.913 × 10^-11^ | 3178.290 | 4.345 × 10^-10^ |
| 2.5 | 15 | 1 | 6.212 × 10^-8^ | 3.293 × 10^-10^ | 188.623 | 1.591 × 10^-9^ |
|  |  | 2 | 8.643 × 10^-8^ | 3.250 × 10^-10^ | 265.975 | 1.541 × 10^-9^ |
|  |  | 3 | 6.595 × 10^-8^ | 1.622 × 10^-10^ | 406.611 | 2.865 × 10^-10^ |
|  | 30 | 1 | 1.311 × 10^-7^ | 3.224 × 10^-10^ | 406.687 | 1.640 × 10^-9^ |
|  |  | 2 | 1.087 × 10^-7^ | 2.758 × 10^-10^ | 394.161 | 1.510 × 10^-9^ |
|  |  | 3 | 1.423 × 10^-7^ | 2.503 × 10^-10^ | 568.341 | 1.530 × 10^-9^ |
| 3.0 | 15 | 1 | 4.706 × 10^-8^ | 4.937 × 10^-11^ | 953.087 | 2.705 × 10^-10^ |
|  |  | 2 | 2.519 × 10^-8^ | 2.287 × 10^-12^ | 11013.642 | 4.195 × 10^-10^ |
|  |  | 3 | 3.201 × 10^-8^ | 6.186 × 10^-12^ | 5174.685 | 2.870 × 10^-10^ |
|  | 30 | 1 | 1.254 × 10^-7^ | 2.662 × 10^-10^ | 470.995 | 1.541 × 10^-9^ |
|  |  | 2 | 1.361 × 10^-7^ | 2.584 × 10^-10^ | 526.612 | 1.567 × 10^-9^ |
|  |  | 3 | 1.316 × 10^-7^ | 2.659 × 10^-10^ | 494.976 | 1.497 × 10^-9^ |
| 3.5 | 15 | 1 | 9.610 × 10^-8^ | 2.912 × 10^-10^ | 329.980 | 1.826 × 10^-9^ |
|  |  | 2 | 7.089 × 10^-8^ | 9.287 × 10^-11^ | 763.325 | 4.605 × 10^-10^ |
|  |  | 3 | 4.441 × 10^-8^ | 3.869 × 10^-11^ | 1148.001 | 1.557 × 10^-9^ |
|  | 30 | 1 | 2.106 × 10^-7^ | 1.218 × 10^-11^ | 17293.668 | 3.840 × 10^-10^ |
|  |  | 2 | 2.075 × 10^-7^ | 1.015 × 10^-11^ | 20440.997 | 2.355 × 10^-10^ |
|  |  | 3 | 2.120 × 10^-7^ | 1.235 × 10^-11^ | 17164.723 | 4.470 × 10^-10^ |
| 4.0 | 15 | 1 | 6.993 × 10^-8^ | 1.958 × 10^-11^ | 3570.837 | 3.835 × 10^-10^ |
|  |  | 2 | 1.429 × 10^-7^ | 1.305 × 10^-11^ | 10952.323 | 3.480 × 10^-10^ |
|  |  | 3 | 3.972 × 10^-8^ | 3.855 × 10^-12^ | 10304.540 | 5.910 × 10^-10^ |
|  | 30 | 1 | 5.243 × 10^-8^ | 1.637 × 10^-10^ | 320.201 | 1.240 × 10^-9^ |
|  |  | 2 | 6.663 × 10^-8^ | 1.058 × 10^-10^ | 629.723 | 5.480 × 10^-10^ |
|  |  | 3 | 2.359 × 10^-8^ | 9.813 × 10^-11^ | 240.439 | 4.680 × 10^-10^ |
| 5.0 | 15 | 1 | 5.905 × 10^-8^ | 1.673 × 10^-10^ | 352.898 | 4.065 × 10^-9^ |
|  |  | 2 | 5.489 × 10^-8^ | 1.583 × 10^-10^ | 346.718 | 3.585 × 10^-9^ |
|  |  | 3 | 4.934 × 10^-8^ | 1.253 × 10^-10^ | 393.709 | 1.109 × 10^-9^ |
|  | 30 | 1 | 1.109 × 10^-7^ | 1.652 × 10^-11^ | 6714.201 | 3.445 × 10^-10^ |
|  |  | 2 | 1.144 × 10^-7^ | 2.186 × 10^-11^ | 5233.356 | 2.951 × 10^-9^ |
|  |  | 3 | 9.181 × 10^-8^ | 1.614 × 10^-11^ | 5687.579 | 1.481 × 10^-9^ |

**Table S2. Mean values and standard deviations of |*I*_on_|, |*I*_off_|, |*I*_on_| / *|I*_off_|, and maximum |*I*_G_| for PAN-OSTs at different PAN concentrations and annealing times (n = 3).**

| PAN  Concentration  (mg/mL) | Annealing  (min) | *\|I*_on_\|  (A) | *\|I*_off_\|  (A) | *\|I*_on_\|/*\|I*_off_\| | Max *\|I*_G_\|  (A) |
| --- | --- | --- | --- | --- | --- |
| 0 | — | 3.230 × 10^-7^  ± 1.328 × 10^-8^ | 1.626 × 10^-7^  ± 2.581 × 10^-9^ | 1.987  ± 0.09488 | 3.119 × 10^-8^  ± 1.185 × 10^-8^ |
| 2.0 | 15 | 9.393 × 10^-8^  ± 2.406 × 10^-8^ | 1.720 × 10^-10^  ± 1.287 × 10^-10^ | 767.3  ± 542.9 | 7.675 × 10^-10^  ± 7.633 × 10^-10^ |
|  | 30 | 7.381 × 10^-8^  ± 2.243 × 10^-8^ | 5.621 × 10^-11^  ± 6.451 × 10^-11^ | 2393  ± 1412 | 4.478 × 10^-10^  ± 5.522 × 10^-11^ |
| 2.5 | 15 | 7.150 × 10^-8^  ± 1.307 × 10^-8^ | 2.722 × 10^-10^  ± 9.526 × 10^-11^ | 287.1  ± 110.5 | 1.140 × 10^-9^  ± 7.391 × 10^-10^ |
|  | 30 | 1.274 × 10^-7^  ± 1.711 × 10^-8^ | 2.828 × 10^-10^  ± 3.656 × 10^-11^ | 456.4  ± 97.15 | 1.560 × 10^-9^  ± 7.000 × 10^-11^ |
| 3.0 | 15 | 3.475 × 10^-8^  ± 1.119 × 10^-8^ | 1.928 × 10^-11^  ± 2.613 × 10^-11^ | 5714  ± 5052 | 3.257 × 10^-10^  ± 8.168 × 10^-11^ |
|  | 30 | 1.310 × 10^-7^  ± 5.372 × 10^-9^ | 2.635 × 10^-10^  ± 4.419 × 10^-12^ | 497.5  ± 27.90 | 1.535 × 10^-9^  ± 3.538 × 10^-11^ |
| 3.5 | 15 | 7.047 × 10^-8^  ± 2.585 × 10^-8^ | 1.409 × 10^-10^  ± 1.329 × 10^-10^ | 747.1  ± 409.3 | 1.281 × 10^-9^  ± 7.233 × 10^-10^ |
|  | 30 | 2.100 × 10^-7^  ± 2.303 × 10^-9^ | 1.156 × 10^-11^  ± 1.224 × 10^-12^ | 18300  ± 1855 | 3.555 × 10^-10^  ± 1.086 × 10^-10^ |
| 4.0 | 15 | 8.418 × 10^-8^  ± 5.305 × 10^-8^ | 1.216 × 10^-11^  ± 7.900 × 10^-12^ | 8276  ± 4088 | 4.408 × 10^-10^  ± 1.313 × 10^-10^ |
|  | 30 | 4.755 × 10^-8^  ± 2.193 × 10^-8^ | 1.225 × 10^-10^  ± 3.585 × 10^-11^ | 396.8  ± 205.6 | 7.520 × 10^-10^  ± 4.245 × 10^-10^ |
| 5.0 | 15 | 5.443 × 10^-8^  ± 4.872 × 10^-9^ | 1.503 × 10^-10^  ± 2.211 × 10^-11^ | 364.4  ± 25.53 | 2.920 × 10^-9^  ± 1.586 × 10^-9^ |
|  | 30 | 1.057 × 10^-7^  ± 1.216 × 10^-8^ | 1.817 × 10^-11^  ± 3.198 × 10^-12^ | 5878  ± 758.6 | 1.592 × 10^-9^  ± 1.307 × 10^-9^ |

**Table S3. *V*_on_ and *μ*_sat_ of PAN-OSTs at different PAN concentrations and annealing times. Data are presented as mean ± SD (n = 3).**

| PAN  Concentration  (mg/mL) | Annealing  (min) | Device | *V*_on_  (V) | *V*_on_  Mean ± SD | *μ*_sat_  (cm^2^V^-1^s^-1^) | *μ*_sat_  Mean ± SD  n = 3 |
| --- | --- | --- | --- | --- | --- | --- |
| 0 | — | 1 | 5.000 | 5.000 | 5.498 × 10⁻³ | 4.955 × 10⁻³ ± 4.750 × 10⁻⁴ |
|  |  | 2 | 5.000 |  | 4.748 × 10⁻³ |  |
|  |  | 3 | 5.000 |  | 4.618 × 10⁻³ |  |
| 2.0 | 15 | 1 | 1.000 | 1.189 ± 0.271 | 0.561 | 0.689 ± 0.117 |
|  |  | 2 | 1.500 |  | 0.791 |  |
|  |  | 3 | 1.067 |  | 0.716 |  |
|  | 30 | 1 | 1.533 | 1.278 ± 0.236 | 1.241 | 1.228 ± 0.186 |
|  |  | 2 | 1.067 |  | 1.036 |  |
|  |  | 3 | 1.233 |  | 1.408 |  |
| 2.5 | 15 | 1 | 0.133 | 0.444 ± 0.278 | 0.725 | 0.612 ± 0.144 |
|  |  | 2 | 0.533 |  | 0.660 |  |
|  |  | 3 | 0.667 |  | 0.450 |  |
|  | 30 | 1 | 1.433 | 1.500 ± 0.371 | 0.677 | 0.612 ± 0.064 |
|  |  | 2 | 1.167 |  | 0.550 |  |
|  |  | 3 | 1.900 |  | 0.610 |  |
| 3.0 | 15 | 1 | 0.500 | 1.233 ± 0.734 | 0.317 | 0.433 ± 0.140 |
|  |  | 2 | 1.967 |  | 0.589 |  |
|  |  | 3 | 1.233 |  | 0.392 |  |
|  | 30 | 1 | 1.500 | 1.633 ± 0.134 | 0.415 | 0.506 ± 0.125 |
|  |  | 2 | 1.767 |  | 0.454 |  |
|  |  | 3 | 1.633 |  | 0.648 |  |
| 3.5 | 15 | 1 | 1.433 | 1.300 ± 0.231 | 0.197 | 0.220 ± 0.035 |
|  |  | 2 | 1.433 |  | 0.202 |  |
|  |  | 3 | 1.033 |  | 0.260 |  |
|  | 30 | 1 | 2.233 | 2.166 ± 0.208 | 0.683 | 0.743 ± 0.452 |
|  |  | 2 | 1.933 |  | 0.325 |  |
|  |  | 3 | 2.333 |  | 1.222 |  |
| 4.0 | 15 | 1 | 1.333 | 1.211 ± 0.397 | 0.182 | 0.373 ± 0.181 |
|  |  | 2 | 1.533 |  | 0.393 |  |
|  |  | 3 | 0.767 |  | 0.543 |  |
|  | 30 | 1 | 0.600 | 1.855 ± 1.142 | 0.373 | 0.710 ± 0.770 |
|  |  | 2 | 2.833 |  | 1.591 |  |
|  |  | 3 | 2.133 |  | 0.165 |  |
| 5.0 | 15 | 1 | 0.600 | 0.589 ± 0.084 | 0.298 | 0.193 ± 0.096 |
|  |  | 2 | 0.667 |  | 0.170 |  |
|  |  | 3 | 0.500 |  | 0.110 |  |
|  | 30 | 1 | 0.933 | 0.755 ± 0.168 | 0.238 | 0.585 ± 0.322 |
|  |  | 2 | 0.733 |  | 0.873 |  |
|  |  | 3 | 0.600 |  | 0.644 |  |

**Table S4. Comparison of event energy consumption in reported synaptic transistor devices.**

| Semiconductor | Dielectric | *V*_D_ | Event energy | Reference |
| --- | --- | --- | --- | --- |
| IGZO | GO-PVA / PVA | 0.5 V | 0.32 fJ | [23] |
| IGZO | PAAm Gel | 0.1 mV | 0.36 fJ | [24] |
| IGZO | PVP | 0.1 mV | 65.75 aJ | [25] |
| ITO | HfLaO | 0.1 V | 93.1 aJ | [26] |
| Pentacene | P(VDF-TrFE-CFE)/PVK | — | 0.75 nJ | [27] |
| PDVT-10 | SiO_2_ / PVN | — | 2.31 nJ | [28] |
| P3HT | Ion Gel | — | 123 uJ | [29] |
| CNTs | PUU | −0.1 V | 44.7 fJ | [30] |
| DPP-DTT | PVDF-HFP | 1 uV | 92 aJ | [31] |
| PEDOT:PSS | PDADMAC | 0.1 V | 2 nJ | [32] |
| WSe_2_ | PEO:LiClO_4_ | 0.1 V | 30 fJ | [33] |
| **PDVT-10** | **SiO_2_ / PAN** | **−0.1 V** | **50 aJ** | **This work** |

**Table S5. Biexponential fitting parameters for EPSC decay under different pulse widths.**

| **Pulse width** | $A_{\mathrm{fast}}$ | $\tau_{\mathrm{fast}}$ | $A_{\mathrm{slow}}$ | $\tau_{\mathrm{slow}}$ | $\Delta I_{\mathrm{res}}$ | $R^{2}$ |
| --- | --- | --- | --- | --- | --- | --- |
| 1 s | 37.51 nA | 0.096 s | 3.16 nA | 1.57 s | ≈ 0 | 0.9971 |
| 2 s | 13.91 nA | 0.210 s | 3.78 nA | 3.23 s | ≈ 0 | 0.9953 |
| 3 s | 60.44 nA | 0.156 s | 6.67 nA | 3.86 s | 0.421 nA | 0.9868 |

**Table S6. Hyperparameters used for PAN reservoir based spoken digit recognition.**

| Parameter | Value | | |
| --- | --- | --- | --- |
| Dataset | FSDD | | |
| Classes | Spoken digits 0-9 | | |
| Total samples | 3000 | | |
| Test samples | 600 | | |
| Spectrogram dimensions, $T\times F$ | 64×64 | | |
| Number of masks, $N_{m}$ | 10 | | |
| Pulse inputs per mask at each time step, $P$ | 15 | | |
| Virtual nodes per mask, $K$ | 35 | | |
| Normalized pulse width range, $\left[ T_{\min} , T_{\mathrm{cycle}} \right]$ | [0.15, 1.0] | | |
| Reservoir dynamics | | Eqs. S29-32 |  |
| Readout | Ridge linear readout ($\lambda=100$) | | |
| Independent runs | 10 | | |
| Test accuracy (mean ± SD) | 96.35 ± 0.46% | | |
| Maximum test accuracy | 97.17% | | |

**Table S7. Test accuracy of spoken digit recognition over ten independent runs.**

| Run index | Seed | Test accuracy (%) | Run index | Seed | Test accuracy (%) |
| --- | --- | --- | --- | --- | --- |
| 1 | 11 | 96.17 | 6 | 66 | 96.33 |
| 2 | 22 | 96.67 | 7 | 77 | 96.17 |
| 3 | 33 | 96.33 | 8 | 88 | 95.50 |
| 4 | 44 | 96.33 | 9 | 99 | 97.17 |
| 5 | 55 | 96.00 | 10 | 110 | 96.83 |
| Test accuracy (mean ± SD) | | | 96.35 ± 0.46% | | |

**Table S8. Comparison of the spoken digit recognition performance based on various neuromorphic devices.**

| Materials | Recognition accuracy | Spoken-digit database | Algorithm | Ref. |
| --- | --- | --- | --- | --- |
| P3CT / Transistor | 88.90% | NIST TI-46 | SNN | [34] |
| WO_x_ / Memristor | 99.20% | NIST TI-46 | RC | [35] |
| TiO_x_ / LPON / Memristor | 94.40% | NIST TI-46 | RC | [36] |
| Sulfonated polyaniline network | 70.00% | FSDD | RC | [37] |
| AgI nanowire network | ~90% | FSDD | RC | [38] |
| Percolating nanoparticle network | >90% | FSDD | RC | [12] |
| P3gCPDT-1gT2/Transistor | 90.70% | FSDD | RC | [39] |
| **PAN / PDVT-10 / Transistor** | **97.17%** | **FSDD** | **RC** | **This work** |

**Note:** SNN: Spike neural network. RC: reservoir computing. FSDD: Free-spoken-digit-dataset.

**Table S9. Memory capacity results of the PAN reservoir. Data are presented as mean ± SD (n = 30).**

| Task | Train MC | Test MC |
| --- | --- | --- |
| STM | 6.74 ± 0.75 | 2.54 ± 0.37 |
| PC/XOR | 6.72 ± 0.49 | 2.57 ± 0.42 |

**Table S10. Parameters and performance summary for speech emotion recognition.**

| Parameter | Value |
| --- | --- |
| Dataset | RAVDESS |
| Emotion classes | 4 (neutral, happy, angry, and sad) |
| Acoustic features | MFCC + ∆ + $\Delta^{2}$ |
| Number of MFCC coefficients, $n_{\mathrm{MFCC}}$ | 50 |
| Input feature dimensions, $T\times F$ | 128 × 150 |
| Number of masks,$N_{m}$ | 4 |
| Pulse inputs per mask at each time step, $P$ | 5 |
| Virtual nodes per mask, $K$ | 64 |
| Reservoir dynamics | Eqs. S29-32 |
| Readout | Ridge regression |
| Regularization coefficient, $\lambda$ | 1×10^−5^ |
| Number of independent runs | 10 |
| Mean test accuracy | 87.0 ± 4.6% |
| Maximum test accuracy | 94.8% |
| Baseline | MFCC-only |
| MFCC-only baseline accuracy | 49.2 ± 16.0% |
| Improvement over baseline | 37.8 percentage points |

**Table S11. Parameters for PAN reservoir assisted snoring detection.**

| Parameter | Value |
| --- | --- |
| Dataset | Kaggle snoring dataset |
| Number of classes | 2 (snoring and non-snoring) |
| Input representation | Binarized mel-spectrogram |
| Mel-spectrogram dimensions | 64 × 62 |
| Binary pixels per input code | Yes |
| Pixels per input group | 4 |
| Number of 4-bit codes per sample | 992 |
| Reservoir dynamics | Eqs. S29-32 |
| Readout | Device-aware 992 × 2 readout |
| Readout weight source | Measured set/reset current updates |
| Number of runs with different random seeds | 5 |

**Table S12. Confusion-matrix outcomes at the best test epoch across five seeds for only baseline and PAN reservoir.**

| Condition | Seed | Best test epoch | TP | TN | FP | FN |
| --- | --- | --- | --- | --- | --- | --- |
| Only baseline | 0 | 188 | 102 | 110 | 40 | 48 |
| Only baseline | 1 | 199 | 98 | 104 | 46 | 52 |
| Only baseline | 2 | 184 | 113 | 98 | 52 | 37 |
| Only baseline | 3 | 169 | 108 | 103 | 47 | 42 |
| Only baseline | 4 | 182 | 99 | 109 | 41 | 51 |
| Pooled | — | — | 520 | 524 | 226 | 230 |
| PAN reservoir | 0 | 180 | 115 | 121 | 29 | 35 |
| PAN reservoir | 1 | 76 | 113 | 123 | 27 | 37 |
| PAN reservoir | 2 | 139 | 116 | 117 | 33 | 34 |
| PAN reservoir | 3 | 149 | 116 | 125 | 25 | 34 |
| PAN reservoir | 4 | 182 | 108 | 124 | 26 | 42 |
| Pooled | — | — | 568 | 610 | 140 | 182 |

**Table S13. Pooled confusion-matrix counts and row-normalized values for snoring detection with only baseline and PAN reservoir.**

| Condition | True class | Predicted non-snoring | Predicted snoring |
| --- | --- | --- | --- |
| **Only baseline** | Non-snoring | **TN = 524 (0.699)** | **FP = 226 (0.301)** |
| **Only baseline** | Snoring | **FN = 230 (0.307)** | **TP = 520 (0.693)** |
| **PAN reservoir** | Non-snoring | **TN = 610 (0.813)** | **FP = 140 (0.187)** |
| **PAN reservoir** | Snoring | **FN = 182 (0.243)** | **TP = 568 (0.757)** |

**Table S14. Classification metrics at the best-test epoch across five independent runs for only baseline and the PAN reservoir.**

| Condition | seed | Best epoch | Accuracy | Balanced accuracy | Precision | Recall | F1 score |
| --- | --- | --- | --- | --- | --- | --- | --- |
| Only baseline | 0 | 188 | 70.67% | 70.67% | 71.83% | 68.00% | 69.86% |
| Only baseline | 1 | 199 | 67.33% | 67.33% | 68.06% | 65.33% | 66.67% |
| Only baseline | 2 | 184 | 70.33% | 70.33% | 68.48% | 75.33% | 71.75% |
| Only baseline | 3 | 169 | 70.33% | 70.33% | 69.68% | 72.00% | 70.82% |
| Only baseline | 4 | 182 | 69.33% | 69.33% | 70.71% | 66.00% | 68.28% |
| PAN reservoir | 0 | 180 | 78.67% | 78.67% | 79.86% | 76.67% | 78.23% |
| PAN reservoir | 1 | 76 | 78.67% | 78.67% | 80.71% | 75.33% | 77.93% |
| PAN reservoir | 2 | 139 | 77.67% | 77.67% | 77.85% | 77.33% | 77.59% |
| PAN reservoir | 3 | 149 | 80.33% | 80.33% | 82.27% | 77.33% | 79.73% |
| PAN reservoir | 4 | 182 | 77.33% | 77.33% | 80.60% | 72.00% | 76.06% |

**Note:** because each test set contained equal numbers of snoring and non-snoring samples (150 samples per class), accuracy and balanced accuracy were numerically identical in the present analysis.

**Table S15. Performance comparison of snoring detection between only baseline and PAN reservoir.**

| Metric | Only baseline | PAN reservoir | Improvement |
| --- | --- | --- | --- |
| Balanced accuracy | 69.60 ± 1.36% | 78.53 ± 1.17% | +8.93 pp |
| Precision | 69.75 ± 1.56% | 80.26 ± 1.61% | +10.51 pp |
| Recall | 69.33 ± 4.24% | 75.73 ± 2.24% | +6.40 pp |
| F1 score | 69.47 ± 2.03% | **77.91 ± 1.32%** | **+8.44 pp** |
| Best individual test accuracy | 70.67% (epoch 188) | 80.33% (epoch 149) | +9.66 pp |

**References**

[1] B. G. Colvin and P. Storr, “The Crystal Structure of Polyacrylonitrile,” *European Polymer Journal* 10 (1974): 337–340, <https://doi.org/10.1016/0014-3057(74)90147-5>.

[2] K. Chen, H. Hu, I. Song, et al., “Organic Optoelectronic Synapse Based on Photon-Modulated Electrochemical Doping,” *Nature Photonics* 17 (2023): 629–637, <https://doi.org/10.1038/s41566-023-01232-x>.

[3] L. Lan, B. Huang, Y. Li, et al., “Stretchable Optoelectronic Synapses with Ultraviolet to Near-Infrared Perception for Retina-Inspired Computing and Vision-Adaptive Sensing,” *npj Flexible Electronics* 9 (2025): 16, https://doi.org/10.1038/s41528-025-00390-y.

[4] S. R. Joshi, S. Lee, and S. Kim, “Semiconducting and Environmentally Responsive Melanin-Doped Silk Nanofibers for Multifunctional Electronic Tattoos,” *Materials Horizons* 12 (2025): 4444–4456, https://doi.org/10.1039/D4MH01929F.

[5] S. G. Noyce, J. L. Doherty, S. Zauscher, and A. D. Franklin, “Understanding and Mapping Sensitivity in MoS₂ Field-Effect-Transistor-Based Sensors,” *ACS Nano* 14 (2020): 11637–11647, https://doi.org/10.1021/acsnano.0c04192.

[6] Z. Dang, F. Guo, Y. Zhao, K. Jin, W. Jie, and J. Hao, “Ferroelectric Modulation of ReS₂-Based Multifunctional Optoelectronic Neuromorphic Devices for Wavelength-Selective Artificial Visual System,” *Advanced Functional Materials* 34 (2024): 2400105, https://doi.org/10.1002/adfm.202400105.

[7] C. Lu, K. Xu, J. Liu, et al., “Accelerating Spiking Neural Networks with Photonic Reconfigurable Devices,” *Nature Communications* 17 (2026): 5367, https://doi.org/10.1038/s41467-026-72119-y.

[8] J. Ming, R. Wang, J. Fu, et al., “Intrinsic Gradient Oxygen-Driven Second-Order Memristors for Continual Reinforcement Learning,” *Nature Communications* 17 (2026): 3367, https://doi.org/10.1038/s41467-026-70014-0.

[9] M. Huang, M. Schwacke, M. Onen, J. del Alamo, J. Li, and B. Yildiz, “Electrochemical Ionic Synapses: Progress and Perspectives,” *Advanced Materials* 35 (2023): 2205169, https://doi.org/10.1002/adma.202205169.

[10] S. Wang, X. Chen, C. Zhao, et al., “An Organic Electrochemical Transistor for Multi-Modal Sensing, Memory and Processing,” *Nature Electronics* 6 (2023): 281–291, https://doi.org/10.1038/s41928-023-00950-y.

[11] S. Seo, B. Kim, D. Kim, et al., “The Gate Injection-Based Field-Effect Synapse Transistor with Linear Conductance Update for Online Training,” *Nature Communications* 13 (2022): 6431, https://doi.org/10.1038/s41467-022-34178-9.

[12] J. B. Mallinson, J. K. Steel, Z. E. Heywood, S. J. Studholme, P. J. Bones, and S. A. Brown, “Experimental Demonstration of Reservoir Computing with Self-Assembled Percolating Networks of Nanoparticles,” *Advanced Materials* 36 (2024): 2402319, https://doi.org/10.1002/adma.202402319.

[13] Y. Zhong, J. Tang, X. Li, B. Gao, H. Qian, and H. Wu, “Dynamic Memristor-Based Reservoir Computing for High-Efficiency Temporal Signal Processing,” *Nature Communications* 12 (2021): 408, https://doi.org/10.1038/s41467-020-20692-1.

[14] L. Appeltant, M. C. Soriano, G. Van der Sande, et al., “Information Processing Using a Single Dynamical Node as Complex System,” *Nature Communications* 2 (2011): 468, https://doi.org/10.1038/ncomms1476.

[15] Z. Chen, W. Li, Z. Fan, et al., “All-Ferroelectric Implementation of Reservoir Computing,” *Nature Communications* 14 (2023): 3585, <https://doi.org/10.1038/s41467-023-39371-y>.

[16] H. Wang, J. Hu, Y. Baek, et al., “Optical Next Generation Reservoir Computing,” *Light: Science & Applications* 14 (2025): 245, https://doi.org/10.1038/s41377-025-01927-6.

[17] K. Toprasertpong, E. Nako, Z. Wang, R. Nakane, M. Takenaka, and S. Takagi, “Reservoir Computing on a Silicon Platform with a Ferroelectric Field-Effect Transistor,” *Communications Engineering* 1 (2022): 21, https://doi.org/10.1038/s44172-022-00021-8.

[18] S. Watt, M. Kostylev, A. B. Ustinov, and B. A. Kalinikos, “Implementing a Magnonic Reservoir Computer Model Based on Time-Delay Multiplexing,” *Physical Review Applied* 15 (2021): 064060, https://doi.org/10.1103/PhysRevApplied.15.064060.

[19] Y. Guo, W. Duan, X. Liu, et al., “Generative Complex Networks within a Dynamic Memristor with Intrinsic Variability,” *Nature Communications* 14 (2023): 6134, https://doi.org/10.1038/s41467-023-41921-3.

[20] M.-K. Lee and M. Mochizuki, “Reservoir Computing with Spin Waves in a Skyrmion Crystal,” *Physical Review Applied* 18 (2022): 014074, https://doi.org/10.1103/PhysRevApplied.18.014074.

[21] W. Lei, L. G. A. Alves, and L. A. N. Amaral, “Forecasting the Evolution of Fast-Changing Transportation Networks Using Machine Learning,” *Nature Communications* 13 (2022): 4252, https://doi.org/10.1038/s41467-022-31911-2.

[22] S. Hou, W. Ma, and X. Zhou, “FastCCC: A Permutation-Free Framework for Scalable, Robust, and Reference-Based Cell-Cell Communication Analysis in Single Cell Transcriptomics Studies,” *Nature Communications* 16 (2025): 11428, https://doi.org/10.1038/s41467-025-66272-z.

[23]	X. Fu, Z. Liang, W. Shuai, et al., “Bend-Resistant and Energy-Friendly GO-PVA/PVA Polymer Electret Synaptic Transistors for Neuromorphic Computations,” *Advanced Functional Materials* 33 (2023): 2308127, <https://doi.org/10.1002/adfm.202308127>.

[24]	B. Huang, C. Deng, L. Lan, et al., “Skin-Like n-Type Stretchable Synaptic Transistors with Low Energy Consumption and Highly Reliable Plasticity for Brain-Inspired Computing,” *Nano Energy* 128 (2024): 109891, <https://doi.org/10.1016/j.nanoen.2024.109891>.

[25]	B. Huang, J. Pan, L. Lan, et al., “Emotion-Modulated Visual Perception in Ultra-Low-Energy Flexible Neuromorphic Synaptic Transistors Enabled by Directionally Polarized Lactam-Based Polymer Electrets,” *Nano Energy* 142 (2025): 111202, <https://doi.org/10.1016/j.nanoen.2025.111202>.

[26]	Y. Liu, T. Wang, K. Xu, et al., “Low-Power and High-Speed HfLaO-Based FE-TFTs for Artificial Synapse and Reconfigurable Logic Applications,” *Materials Horizons* 11 (2024): 490–498, <https://doi.org/10.1039/D3MH01461D>.

[27]	Y. Bu, T. Xu, S. Geng, S. Fan, Q. Li, and J. Su, “Ferroelectrics-Electret Synergetic Organic Artificial Synapses with Single-Polarity Driven Dynamic Reconfigurable Modulation,” *Advanced Functional Materials* 33 (2023): 2213741, <https://doi.org/10.1002/adfm.202213741>.

[28]	R. Yu, E. Li, X. Wu, et al., “Electret-Based Organic Synaptic Transistor for Neuromorphic Computing,” *ACS Applied Materials & Interfaces* 12 (2020): 15446–15455, <https://doi.org/10.1021/acsami.9b22925>.

[29]	H. Shim, K. Sim, F. Ershad, et al., “Stretchable Elastic Synaptic Transistors for Neurologically Integrated Soft Engineering Systems,” *Science Advances* 5 (2019): eaax4961, <https://doi.org/10.1126/sciadv.aax4961>.

[30]	W. Huang, Y. Wang, Y. Zhang, et al., “Intrinsically Stretchable Carbon Nanotube Synaptic Transistors with Associative Learning Ability and Mechanical Deformation Response,” *Carbon* 189 (2022): 386–394, <https://doi.org/10.1016/j.carbon.2021.12.081>.

[31]	X. Wang, W. Lu, P. Wei, et al., “Artificial Tactile Recognition Enabled by Flexible Low-Voltage Organic Transistors and Low-Power Synaptic Electronics,” *ACS Applied Materials & Interfaces* 14 (2022): 48948–48959, <https://doi.org/10.1021/acsami.2c14625>.

[32]	Q. Liu, Y. Liu, J. Li, et al., “Fully Printed All-Solid-State Organic Flexible Artificial Synapse for Neuromorphic Computing,” *ACS Applied Materials & Interfaces 11* (2019): 16749–16757, <https://doi.org/10.1021/acsami.9b00226>.

[33]	J. Zhu, Y. Yang, R. Jia, et al., “Ion Gated Synaptic Transistors Based on 2D van der Waals Crystals with Tunable Diffusive Dynamics,” *Advanced Materials* 30 (2018): 1800195, <https://doi.org/10.1002/adma.201800195>.

[34]	S. J. Kim, J.-S. Jeong, H. W. Jang, et al., “Dendritic Network Implementable Organic Neurofiber Transistors with Enhanced Memory Cyclic Endurance for Spatiotemporal Iterative Learning,” *Advanced Materials* 33 (2021): 2100475, <https://doi.org/10.1002/adma.202100475>.

[35]	J. Moon, W. Ma, J. H. Shin, et al., “Temporal Data Classification and Forecasting Using a Memristor-Based Reservoir Computing System,” *Nature Electronics* 2 (2019): 480–487, <https://doi.org/10.1038/s41928-019-0313-3>.

[36]	R. Islam, Y. Shi, G. V. de Oliveira Silva, M. Sachdev, and G.-X. Miao, “Volatile and Nonvolatile Programmable Iontronic Memristor with Lithium Imbued TiOx for Neuromorphic Computing Applications,” *ACS Nano* 18 (2024): 22045–22054, <https://doi.org/10.1021/acsnano.4c05137>.

[37]	Y. Usami, B. van de Ven, D. G. Mathew, et al., “In-Materio Reservoir Computing in a Sulfonated Polyaniline Network,” *Advanced Materials* 33 (2021): 2102688, <https://doi.org/10.1002/adma.202102688>.

[38]	S. Lilak, W. Woods, K. Scharnhorst, et al., “Spoken Digit Classification by In-Materio Reservoir Computing With Neuromorphic Atomic Switch Networks,” *Frontiers in Nanotechnology* 3 (2021): 675792, <https://doi.org/10.3389/fnano.2021.675792>.

[39]	R. Liu, Y. He, X. Zhu, et al., “Hardware-Feasible and Efficient N-Type Organic Neuromorphic Signal Recognition via Reservoir Computing,” *Advanced Materials* 37 (2025): 2409258, <https://doi.org/10.1002/adma.202409258>.
